# Electrophysiological correlates of concept type shifts

**DOI:** 10.1371/journal.pone.0212624

**Published:** 2019-03-05

**Authors:** Natalia Bekemeier, Dorothea Brenner, Anne Klepp, Katja Biermann-Ruben, Peter Indefrey

**Affiliations:** 1 Department of Linguistics, Heinrich Heine University, Düsseldorf, Germany; 2 Institute of Clinical Neuroscience and Medical Psychology, Medical Faculty, Heinrich Heine University, Düsseldorf, Germany; 3 Max Planck Institute for Psycholinguistics and Donders Institute for Brain, Cognition and Behaviour, Nijmegen, The Netherlands; University of Amsterdam, NETHERLANDS

## Abstract

A recent semantic theory of nominal concepts by Löbner [1] posits that–due to their inherent uniqueness and relationality properties–noun concepts can be classified into four concept types (CTs): sortal, individual, relational, functional. For sortal nouns the default determination is indefinite (*a stone*), for individual nouns it is definite (*the sun*), for relational and functional nouns it is possessive (*his ear*, *his father*). Incongruent determination leads to a concept type shift: *his father* (functional concept: unique, relational)–*a father* (sortal concept: non-unique, non-relational). Behavioral studies on CT shifts have demonstrated a CT congruence effect, with congruent determiners triggering faster lexical decision times on the subsequent noun than incongruent ones [2, 3]. The present ERP study investigated electrophysiological correlates of congruent and incongruent determination in German noun phrases, and specifically, whether the CT congruence effect could be indexed by such classic ERP components as N400, LAN or P600. If incongruent determination affects the lexical retrieval or semantic integration of the noun, it should be reflected in the amplitude of the N400 component. If, however, CT congruence is processed by the same neuronal mechanisms that underlie morphosyntactic processing, incongruent determination should trigger LAN or/and P600. These predictions were tested in two ERP studies. In Experiment 1, participants just listened to noun phrases. In Experiment 2, they performed a wellformedness judgment task. The processing of (in)congruent CTs (*his sun* vs. *the sun*) was compared to the processing of morphosyntactic and semantic violations in control conditions. Whereas the control conditions elicited classic electrophysiological violation responses (N400, LAN, & P600), CT-incongruences did not. Instead they showed novel concept-type specific response patterns. The absence of the classic ERP components suggests that CT-incongruent determination is not perceived as a violation of the semantic or morphosyntactic structure of the noun phrase.

## Introduction

Nouns and the concepts they denote are not all the same. Next to the well-known distinction between count and mass nouns, different kinds of count nouns can also be distinguished. Count nouns differ with respect to whether they typically denote something unique (*the sun*, *the Pope*, *my mother*, *my size*) or something that typically comes in or may come in more than one exemplar (*stones*, *legs*, *arguments*, *brothers*). Count nouns also differ with respect to their relationality, that is whether they typically require an argument or not. Whereas a noun like *stone* does not need an argument, nouns such as *mother* and *size* typically are related to some kind of ‘possessor’ argument whose mother or size is referred to in some communicative context. Based on such distinctions, semanticists generally agree on the existence of different categories of noun concepts, although not necessarily on how many categories should be distinguished (for overviews see [4–9]).

Because noun concepts differ with respect to their conceptual properties, and contexts differ with respect to the kinds of properties they require, there can be a mismatch between the context and the noun. As the nouns still need to be interpreted, such mismatches result in ‘type shifts’ or ‘type coercion’ [5, 10, 11]. Mismatches may concern different conceptual properties. Most familiar are probably cases of animacy mismatches as in *stone lion*, where the conceptual feature ‘animate’ of the concept ‘lion’ does not match the requirements of the preceding modifier and must be deleted to interpret the phrase, or “*The ham sandwich from* *Table* 3 *wants to pay*.”, where the verb *pay* requires an animate subject, resulting in a metonymic shift from ‘ham sandwich’ to ‘customer who has the ham sandwich’ [11]. But also a conceptual feature like uniqueness may be subject to mismatch as in the noun phrase *a sun*, where the indefinite determiner requires a non-unique noun, so that the interpretation of the phrase triggers a conceptual shift from the unique ‘sun’ (as in ‘our’ sun) to a non-unique ‘sun’ (as in ‘kind of astronomic body’).

Löbner recently proposed a ‘Theory of Concept Types and Determination’ [1] that differentiates between four concept types (CTs) of nouns. The noun types are categorized according to two binary referential features: uniqueness [U] and relationality [R] (see Table 1). *Sortal* nouns represent the prototypical type of nouns: they are non-unique and non-relational [-U] [-R], i.e. they do not need any possessor specification (e.g. *a stone*, *a lion*). These properties of sortal nouns are reflected in their preferred (default) determination context. Congruent determinations of a sortal noun are indefinite (*a stone*), plural (*stones*), quantificational (*some*, *many stones*), and demonstrative (*this stone*). The referents of *individual* nouns are inherently unique [+U] and also non-relational [-R]. These nouns denote individual terms such as proper names, unique institutions, and unique referents (e.g. *the moon*, *the pope*). They combine per default with singular definite determination. The referents of inherently relational [+R] noun types are characterized in terms of their relationship to another object or entity. *Relational* nouns [-U] [+R] represent non-unique concepts in a context of a given possessor, such as non-unique parts of the body or kinship terms. Congruent determination includes indefinite and plural typically combined with a possessor argument for referential use: e.g. *a brother of my friend*, *his foot*. *Functional* nouns [+U] [+R] denote unique referents with an appropriate possessor in a given context. Therefore, they require both the saturation of the possessor argument and singular definite determination: e.g. *the author of “War and Peace”*, *her mother*, *his nose*.

**Table 1:** Four types of nouns and their respective congruence with modes of determination (from Löbner [1]).

	[-U]	[+U] inherently unique
**[-R]**	SORTAL NOUNS	INDIVIDUAL NOUNS
	*stone book adjective water*	*moon weather date Maria*
	√ Indef., Plural, quantif., dem.	¬ Indef., Plural, quantif., dem.
	¬ singular definite	√ singular definite
	√ absolute	√ absolute
	¬ relational, possessive	¬ relational, possessive
**[+R] inherently relational**	RELATIONAL NOUNS	FUNCTIONAL NOUNS
	*sister leg part attribute*	*father head age subject* (gramm.)
	√ Indef., Plural, quantif., dem.	¬ Indef., Plural, quantif., dem.
	¬ singular definite	√ singular definite
	¬ absolute	¬ absolute
	√ relational, possessive	√ relational, possessive

The concept type of a noun may be shifted by means of coercion, if the requirements of the default, i.e. congruent, determination are not met (e.g. *his stone*, *a moon*, *the brother*, *a nose* [1]. As coercion brings about higher semantic complexity, incongruent determination (i) tends to be less frequent, (ii) tends to receive more salient expression, and (iii) requires contextual support [1].

In the present study, we investigated such CT shifts. We expected that, if concepts are lexically specified for the features *Uniqueness* and *Relationality*, CT-(in)congruent determination might affect lexical retrieval or word recognition, similar to grammatical gender (in)congruent determination (see below). On the other hand, (in)congruent determination might not affect lexical access but rather induce a post-lexical cognitive type shift (coercion) operation [1, 2].

We report two studies that employed the event-related potentials (ERP) technique to study the neural correlates of concept type shifts. The ERP technique is used to record the online brain activity elicited by a certain event (a picture, a syllable, etc.) with millisecond precision. Therefore, this technique is an appropriate tool for the investigation of online processing of CT shifts. In the following sections, we shall first discuss behavioral evidence for the lexical specification of nouns for the conceptual features *Uniqueness* and *Relationality*, and after that, we will describe the ERP components that could be relevant for the investigation of CT shifts.

### Psycholinguistic evidence for a CT-congruence effect

To investigate a possible CT-congruence effect predicted by Löbner’s (1) theory, Brenner and colleagues [2] presented listeners with German noun phrases in an auditory lexical decision experiment employed an approach introduced by Bölte & Connine [12] for the investigation of grammatical gender congruence effects in noun phrases. They combined nouns of the four CTs with congruent determiners, incongruent determiners, or a length-matched noise stimulus (‘no determiner’ or ‘neutral’ condition). The data showed a significant facilitatory CT-congruency effect with congruent determiner-noun combinations resulting in shorter lexical decision times relative to incongruent and no determination. Further analyses of the factors *Uniqueness* and *Relationality* revealed the influence of both factors on the congruency effect. Following the approach by Bölte & Connine [12], Brenner [3] then ran a further experiment using a phoneme monitoring task that selectively taps into lexical retrieval. In this experiment the CT-congruence effect disappeared, suggesting that CT congruence affects a post-lexical rather than a lexical processing stage. Brenner [3], furthermore, conducted analyses of the correlation between the size of the CT-congruence effect and the corpus-based co-occurrence frequencies of the determiner-noun combinations. There was no significant correlation for any of the four concept types, suggesting that the CT-congruence effect does not reflect some kind of familiarity assessment. Brenner et al. [2, 3], therefore, propose a facilitatory influence of congruent uniqueness and relationality features on the post-lexical build-up of noun phrases. Note, however, that the behavioral evidence for a post-lexical locus of the CT congruence effect was indirect, relying on the presence and absence of the effect in two different tasks. We, therefore, decided to investigate CT congruence with the ERP technique. This method has a high temporal resolution and thus might provide direct evidence about the time course of the CT congruence effect. In addition, ERPs might be informative about the nature of the effect, if the ERP signature of CT incongruences would be similar to known signatures of semantic or syntactic incongruences.

### Semantic and syntactic ERP components

There are several ‘classic’ ERP components, such as N400 and P600 [13–23] that have been extensively studied, well-documented and used as an index of semantic and syntactic processing. The best explored ERP component is the N400, which is a negative-going deflection observed 300–500 ms post-stimulus over the central and central-parietal electrode sites, and peaking around 400 ms [13, 14, 16, 17, 24]. This ERP component is known to reflect various aspects of and difficulties in semantic processing, such as word frequency [25], priming [26–29], repetition [30, 31], lexical status of the stimuli [17, 32–35], etc. These factors demonstrate that the N400 can tap into lexical access. However, the N400 is also sensitive to the post-lexical factors, such as cloze probability, expectancy, and semantic anomaly [16, 36–38]. Being sensitive to semantic congruence, the N400 component could be a useful tool for the investigation of CT shifts. If matching the referential features [U] and [R] between a determiner and a noun is similar to selectional restrictions, congruent determination should facilitate lexical retrieval, indexed by the absence of N400. Incongruent determination, on the other hand, should elicit an N400 effect.

Left anterior negativity (LAN) is an ERP component that is observed within the same latency as the N400, i.e. 300–500 ms post-stimulus, however with a distinct topography and functionality (for an alternative account see Tanner and Van Hell [39]). As the label follows, this ERP component is a negativity usually observed at left anterior or frontal electrode sites [40, 41]. The LAN was reported to be sensitive to morphosyntactic violations, such as pronoun case violation [42], violation of inflection [43–46], subject-verb agreement violation [42, 47–52], and gender agreement violations [53, 54]. The LAN reflects (morpho)syntactic processing that is restricted to rather local syntactic structures. If the conflict in parsing triggered by the local violation is grave enough, the LAN can be followed by another syntactic ERP component, viz. the P600. The P600 is a positive-going deflection observed around 500–900 ms post-stimulus at central-parietal sites [19, 22, 55, 56]. The P600 can occur on its own or can follow the LAN or the N400. This ERP component is sensitive to the higher-order cognitive processing, such as parsing and repair. It was reported in the studies exploring the garden path effect [55, 57], subject-verb number and word order constraint violation [22], syntactic complexity [58, 59], violation of a strong contextual constraint [60–63], etc.

Several ERP studies that investigated number and gender agreement violations reported a biphasic LAN-P600 pattern [53, 54, 64, 65]. Barber and Carreiras [54] explored whether mental representations of grammatical gender and number have an impact on the syntactic processing in reading. Agreement violations in word pairs triggered an N400 effect in noun-adjective combinations and an additional LAN in article-noun combinations. The same words inserted in sentences elicited a biphasic LAN–P600 pattern. The P600 effect in the late latency was more prominent for gender than for number violations. The authors interpreted the results of the study in favor of the hypothesis that grammatical gender should be stored in the lexical representation as opposed to number that should combine with the word stem via application of a morphological rule. Molinaro et al. [53] also observed a biphasic LAN-P600 pattern. In his study, this pattern was triggered by phonotactic and grammatical gender agreement violations in Spanish sentences. Loerts and colleagues investigated gender agreement violations in spoken Dutch sentences [66]. Here, the critical noun had either a low or high cloze probability within the sentence context. Whereas low cloze nouns elicited an N400 effect, independent of gender violations, there was an interaction between the cloze probability and gender mismatch in the early latency of the P600 component (500–550 ms): high cloze nouns with a gender mismatch triggered an earlier P600 component relative to low cloze items. Both high and low cloze nouns with a gender mismatch elicited similar P600 effects in the late time window (up to 1500 ms). The results of Loerts et al. (2013) study suggest that the onset of the P600 component could be affected by such factors as semantic expectancy.

### Rationale of the present study

The objective of the present study was to establish the neural correlates of concept type shifts, more specifically whether the CT congruence effect could be indexed by classic ERP components like N400, LAN or P600 or a novel electrophysiological signature specific to CT incongruences. We, therefore, presented our participants with spoken CT congruent and incongruent determiner-noun phrases of all four concept types of Löbner (1). To show that our paradigm was sensitive enough to detect the classic effects and to be able to compare them with potential CT congruence effects, we included control conditions that were likely to yield the classic effects. In these control conditions we presented three kinds of nominal phrases consisting of an adjective and a noun: (i) Correct phrases; (ii) Semantic violation phrases with a semantic mismatch between the adjective and the noun to elicit an N400 effect; and (iii) Morphosyntactic violation phrases with a gender mismatch between the adjective and the noun to elicit a LAN and/or P600 effect.

We reasoned that, if (in)congruent determination affects the lexical retrieval of the noun or inhibits the semantic integration of the noun with the preceding determination, it should be reflected in the amplitude of the N400 component. If the mapping of uniqueness and relationality features between a determiner and a noun follows grammatical combinability rules, incongruent determination should trigger a (morpho)syntactic violation brain response, i.e. LAN and/or P600.

We did not have a specific hypothesis on whether potential electrophysiological correlates of CT incongruences (classic or novel) should be of a general nature (across all concept types) or concept-type specific as there were good reasons for both possibilities. A general CT-shift or congruence effect was suggested by the results of hemodynamic studies on compositional semantic processing. A recent meta-analysis [67] of a large number of hemodynamic studies on sentence level semantic processing showed a common neural substrate of semantic violations, semantic ambiguities and different kinds of conceptual shifts (metaphor, metonymy, irony) in anterior-inferior Broca’s area (BA 45/47). This finding suggests the existence of neuronal populations supporting combinatorial semantic processing in general. A more specific reason to expect a common electrophysiological effect across the four concept types was the observation in the behavioral experiments [2, 3] that both uniqueness and relationality congruence contributed to the overall CT-congruence effect. On the other hand, the conceptual features underlying the incongruence differed between the four concept types. For sortal nouns there was a uniqueness mismatch, for individual and relational nouns a relationality mismatch, and for functional nouns both a uniqueness and relationality mismatch. As the neural mechanisms underlying uniqueness and relationality shifts are not known and quite possibly distinct given the different nature of the operation involved (establishing/deleting uniqueness or a possessor argument) it was clearly possible that the two features might be differentially indexed by the classic ERP components (e.g. one having a quasi-grammatical status the other not) and that in consequence incongruences of the different concept types might be differentially indexed too.

## Experiment 1

### Methods

#### Participants

Twenty-five students of the Heinrich-Heine-University, Düsseldorf (12 male, age range: 19–33 years, mean: 25.21, SD: 3.7) took part in the study. One participant had to be excluded due to excessive muscular artifacts. The participants were native speakers of Standard German (no other language learned before the age of five) and were assessed right-handed by the Edinburgh handedness test [68]. They had normal or corrected-to-normal vision, were not taking any psychoactive medication, reported no hearing impairments, and no psychological or neurological disorders. The participants signed an informed consent form and were paid 16 € for participation. The study was conducted in compliance with the Declaration of Helsinki and was approved by the Ethics Committee of the Medical Faculty of the Heinrich-Heine-University, Düsseldorf, Germany; study number: 5822R.

#### Materials

Experimental items consisted of eight sets of determiner + noun (DN) phrases for the concept type (CT) conditions and three sets of adjective + noun (AN) phrases (see Table 2 for examples). The congruent CT conditions consisted of the four CTs with their respective default determination: *sortal congruent (****SC****)* with an indefinite article, *individual congruent (****IC****)* with a definite article, *relational congruent (****RC****)* with a possessive pronoun, and *functional congruent (****FC****)* with a possessive pronoun. In incongruent CT conditions the determiner, though grammatically correct, was only possible within a certain pragmatic context: *sortal incongruent (****SI****)* with a definite article, *individual incongruent (****II****)* with a possessive pronoun, *relational incongruent (****RI****)* with a definite article, *functional incongruent (****FI****)* with an indefinite article. Only relatively frequent words (see Table 2) were selected for the study [69]. The adjective + noun phrases (AN conditions) were created in the following manner: for the *Correct* condition, we chose an adjective that according to the Leipzig Corpus (http://corpora.informatik.uni-leipzig.de/de?corpusId=deu_newscrawl_2011) was a frequently occurring left neighbor of a noun. We then asked five native speakers of German to evaluate the probability of co-occurrence of the adjective-noun pairs. If they rated this probability as low, we asked them to provide an adjective that would increase the probability of occurrence of the given noun. As a result, twelve adjectives were replaced. For the *Semantic* violation condition, we randomly re-assigned the adjectives of the *Correct* condition list to other nouns of the list, and asked our informants to evaluate the semantic compatibility of the resulting adjective + noun combinations. In cases where the novel combinations were judged compatible we again re-assigned the adjectives until all phrases were judged incompatible. For the *(Morpho)Syntactic* violation condition, we changed the gender-marking suffixes of the adjectives of the *Correct* condition list such, that they mismatched the nouns in terms of gender.

**Table 2:** Frequency of occurrence of nouns and various duration values.

Condition	Example	Celex		Leipzig	Probability of the determiner as a left neighbor
		overall	spoken		
**Sortal Congruent**	ein Stein (*a stone)*	437.51	33.11	5967.66	27%
**Sortal Incongruent**	der Stein *(the stone)*				33%
**Individual Congruent**	der Papst *(the pope)*	415.17	28.86	8373.1	32%
**Individual Incongruent**	sein Papst *(his pope)*				0.46%
**Relational Congruent**	sein Ohr *(his ear)*	416.91	28.23	5199.5	10.52%
**Relational Incongruent**	das Ohr *(the ear)*				29%
**Functional Congruent**	seine Mutter *(his mother)*	421.82	33.15	8266.01	6%
**Functional Incongruent**	eine Mutter *(a mother)*				4%
***Nominal Phrase*: *Correct***	interessanter Artikel *(interesting (m*.*) article (m*.*))*	559.72	48.08	10305.7	8.98%
***Nominal Phrase*: *Semantic violation***	koffeinfreier Artikel *(decaffeinated (m*.*) article (m*.*))*				0%
***Nominal Phrase*: *Syntactic violation***	interessantes Artikel *(interesting (n*.*) article (m*.*))*				0%

The first column of the table represents experimental conditions: concept types with congruent and incongruent determination (rows 2–9) and nominal phrases with a congruent (row 10), semantically mismatching (row 11) and gender mismatching (row 12) adjective. Column 2 provides examples of the experimental conditions. Columns 3–5 demonstrate overall and spoken frequency of occurrence in Celex and overall frequency in the Leipzig Corpus. The last column provides the probability of the determiner/adjective as a left neighbor in the Leipzig Corpus.

A male speaker of Standard German (a speech therapist) read the experimental items with a variable intonation for a recording. To avoid habituation to a specific intonation, three prosodic patterns, i.e. tokens, were recorded: a rising, even, and a falling intonation. The resulting 3567 phrases (*1920 CT phrases*: 80 DPs per condition x 8 conditions x 3 tokens; *1647 AN phrases*: 183 NPs per condition x 3 conditions x 3 tokens) were recorded and digitized with 16 bits precision and 44.1 kHz sampling rate using a Marantz PMD620 portable stereo audio recorder. The stimulus materials were processed with the sound editing software Adobe Audition 3.0: the RMS amplitude of the samples was normalized to 70%, the on- and offsets (30 ms) of the samples were processed with “smooth fade in/ fade out” function. Since experimental items varied in length and stress pattern (see Table 3), we set the event marker on the recognition point. The recognition point was established by means of a corpus analysis considering all word candidates with initial phonological overlap. The timing of the event marker was measured for each phrase individually using Adobe Audition 3.0 software.

**Table 3:** Various duration values for the concept type and adjective+noun conditions.

Condition	Recognition point	File length	Determiner	
			Type	Duration
**Sortal Congruent**	402.18 (SD: 87.2 Range: 224–670)	741.89 (SD: 115.5 Range: 484–1083)	ein/eine	200.74 (SD: 42.89 Range: 101–305)
**Sortal Incongruent**	353.59 (SD: 106.7 Range: 178–641)	696.51 (SD: 123.6 Range: 440–1000)	der/die/das	159.86 (SD: 49.50 Range: 79–316)
**Individual Congruent**	420.84 (SD: 99.9 Range: 209–711)	784.27 (SD: 140.6 Range: 480–1300)	der/die/das	161.45 (SD:45.82 Range: 78–299)
**Individual Incongruent**	525.82 (SD: 109.5 Range: 328–808)	887.1 (SD: 152.3 Range:600–1300)	sein/seine	277.80 (SD:52.24 Range: 181–438)
**Relational Congruent**	553.45 (SD: 93.1 Range: 343–841)	889.43 (SD: 132.1 Range: 560–1250)	sein/seine	284.42 (SD:47.72 Range: 176–431)
**Relational Incongruent**	436.44 (SD: 90.01 Range: 250–714)	778.17 (SD: 117.2 Range:492–1065)	der/die/das	171.11 (SD: 49.88 Range: 84–323)
**Functional Congruent**	518.44 (SD: 91.9 Range: 266–712)	892.06 (SD: 28.45 Range: 600–1296)	sein/seine	281.86 (SD: 47.33 Range: 171–466)
**Functional Incongruent**	465.15 (SD: 85.76 Range: 269–669)	841.69 (SD: 124.7 Range: 560–1250)	ein/eine	228.01 (SD: 37.33 Range: 140–344)
***Nominal Phrase*: *Correct***	729.43 (SD: 162.9 Range: 394–1277)	1013.99 (SD: 185.2 Range: 650–1727)	*adj*.	543.89 (SD: 148.6 Range: 246–1100)
***Nominal Phrase*: *Semantic***	718.15 (SD:153.4 Range: 389–1233)	1000.53 (SD: 171.9 Range: 620–1956)	*adj*.	536.19 (SD: 148.4 Range: 237–1013)
***Nominal Phrase*: *Syntactic***	739.95 (SD: 174.5 Range: 327–1290)	1022.7 (SD: 188.7 Range: 600–1573)	*adj*.	562.67 (SD: 154.7 Range: 240–1087)

The first column of the table lists the experimental conditions: concept types with congruent and incongruent determination (rows 2–9) and nominal phrases with a congruent (row 10), semantically mismatching (row 11) and gender mismatching (row 12) adjective. Column 2 provides the timing of the trigger set on the recognition point. The duration of the audio file is given in column 3. The type (column 4) and the duration (column 5) of determiners are demonstrated in the last two columns. The information about duration and timing is provided as a mean value, with a standard deviation and range of duration in brackets.

The stimuli were pseudorandomized to produce twelve lists divided into three experimental runs. Each list consisted of 503 phrases: 320 CT phrases (40 DPs per condition) and 183 AN phrases. The nouns in the phrases were used only once in each list. Every list had a unique combination of tokens of experimental items and phrases never appeared in the same context across lists. Each of the twelve lists was pseudorandomized once again to yield another set of lists so that in result every participant heard an individual list of stimuli.

#### Procedure

The participants were tested individually in a sound-attenuating booth. They were seated in a comfortable chair in front of a computer monitor. Their task was to listen to auditorily presented phrases in blocks of 3–8 items. A visual probe was presented on the computer screen after each auditory block. The visual probes consisted of items that were used in the experimental conditions: 110 phrases = (8 CT conditions + 3 AN conditions) x 10 phrases per condition. The participants were asked to press the right mouse key if the presented phrase had appeared in the last auditory block, and to press the left mouse key if the phrase had not appeared in the last auditory block. Half of the visual probes had occurred in the preceding auditory blocks. In the other half, the noun had occurred, but with a different determiner/adjective. All stimuli were presented using the software package of Presentation (Neurobehavioural Systems, Inc., Albany, CA, USA). The auditory stimuli were presented binaurally through headphones with an intertrial interval of 2 seconds. During the presentation of the auditory stimuli the computer screen was black with a white fixation cross in the middle; the fixation cross appeared 200 ms before the stimulus presentation and disappeared at the offset of the wave file. The participants were instructed to avoid any body or eye movements, to fixate on the cross, but were free to blink when the cross was not displayed during the ISI or during the presentation of the visual probe. Before the experiment, the participants had a short practice block. The whole procedure took approximately 2 hours, including set-up and two 5-minute breaks between the runs.

#### EEG recording

**Fig 1:**
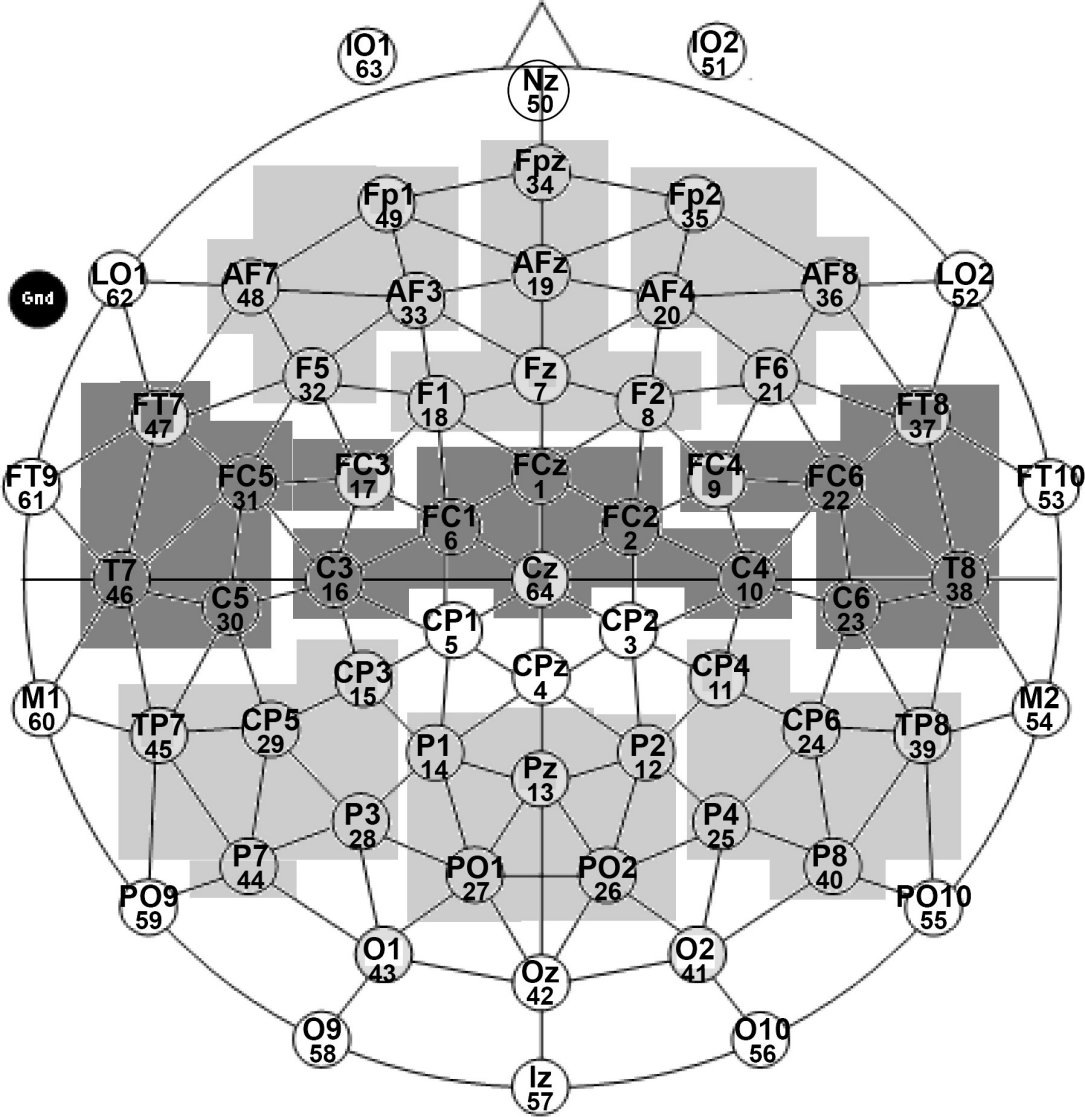
Electrode positions and regions of interest (ROIs). EasyCap 64 channels equidistant, montage M10 (original image adapted with kind permission from EASYCAP GmbH); ROIs are grouped according to two topographical factors, i.e. Anteriority (Anterior/ Central/ Posterior) and Laterality (Left/ Midline/ Right).

The electroencephalogram (EEG; BrainAmp amplifier; Brain Products GmbH, Gilching, Germany) was recorded using 64 sintered Ag/AgCl electrodes placed in an elastic electrode cap (see Fig 1: EasyCap 64 channels equidistant, montage M10) with respect to a vertex reference (Cz). The EEG data underwent an average-reference transformation off-line. The electrooculogram (EOG) was recorded bipolarly using two electrodes positioned near the outer canthi of the eyes (LO1, LO2) for horizontal eye movements (HEOG). Vertical eye movements (VEOG) were monitored with electrodes placed below the eyes (IO1 and IO2) and between the eyebrows (Nz). The ground electrode was affixed to the right cheek. The impedances were kept below 5 kΩ at the scalp sites and below 10 kΩ for the EOG. The EEG was recorded continuously with a sampling rate of 500 Hz, a 0.1 μV resolution, and a low (0.016 Hz) and high (250 Hz) cutoff filter.

#### Data processing

The EEG data were processed using Brain Vision Analyzer 2.1 software (Brain Products GmbH, Gilching, Germany). The raw EEG was filtered with a high-pass filter at 0.1 Hz and a low-pass filter at 30 Hz prior to the ocular correction. The ocular correction was performed with the independent-component analysis (ICA): *Restricted Infomax* algorithm, interval data (0–328 s), with 512 ICA steps. The eye-artifact corrected data were re-referenced to the common average reference and segmented into epochs with a total duration of 1400 ms (a 200-ms baseline and a 1200-ms post-stimulus interval), time-locked to the recognition point of the noun. The epochs within the maximum voltage step of 50 μV/ms and not exceeding the maximum allowed amplitude of 75 μV were averaged for each condition and for each participant, and were baseline corrected (-200-0 ms). The total number of rejected trials was 9.9%.

#### Data analysis

The averaged EEG data were exported from Brain Vision Analyzer into MatLab for data management. All statistical analyses were performed with R 2.7.2 (The R Foundation for Statistical Computing), and SPSS (IBM SPSS Statistics 21, Inc.) software. The time windows that entered statistical analyses were determined according to the literature, however, upon visual observation of the grand average waveforms, we decided to use an additional time window, i.e. **0–200 ms** post recognition point, that demonstrated a prominent difference between the congruent and incongruent CT conditions. The auditory N400 has been reported to have a latency of 200–400 ms post recognition point [13, 28, 70–74], whereas the literature on the auditory LAN component has reported no homogenous modality-specific latency shifts [75–79]. Therefore, two adjacent time windows were used to capture the N400 and LAN effects: **200–350** and **350–500** ms. The last time window (**600–700** ms) was chosen based on the P600 literature [19, 77]. We used the abovementioned latencies in the analyses of both the CT conditions and the AN phrases.

The AN phrases were analyzed separately for each latency with three within-subject factors: *Anteriority* (Anterior/ Temporal/ Posterior), *Laterality* (Left/ Right), and *Condition* (Correct/ Morphosyntactic violation/ Semantic violation) in the first omnibus ANOVA (General Linear Model in SPSS) calculated for the lateral sites (see Fig 1 for the layout and the regions of interest); and with the factors *Anteriority* (Anterior/ Central/ Posterior) and *Condition* in the second omnibus ANOVA (calculated for the midline sites). If the three-way interaction of the type *Anteriority*:*Laterality*:*Condition* in the first analysis and the two-way interaction *Anteriority*:*Condition* at the midline sites analysis reached significance, repeated measures one-way ANOVAs with the factor *Condition* were conducted within each ROI. Paired t-tests were performed if the repeated measures one-way ANOVA reached significance in a given ROI. The reported degrees of freedom and p-values have been adjusted with Greenhouse-Geisser and Bonferroni corrections.

For each latency, two omnibus repeated measures ANOVAs (General Linear Model in SPSS) were conducted in the CT conditions. The first type of omnibus ANOVAs included four within-subject factors: two topographical factors, i.e. *Anteriority* (Anterior/ Temporal/ Posterior) and *Laterality* (Left / Right), and two CT-related factors, i.e. *Congruence* (Congruent/ Incongruent), and *CT* (Individual/ Sortal/ Functional/ Relational). The second omnibus ANOVA was run for the Midline sites with three factors: *Anteriority* (Anterior/ Central/ Posterior), *Congruence*, and *CT*. If the CT-related factors displayed significant interaction with both topographic factors, i.e. *Anteriority*:*Laterality*:*Congruence*:*CT*, we conducted two-way repeated measures ANOVAs with the factors *Congruence* and *CT* in each ROI. If these ANOVAs yielded a significant main effect of *Congruence* and/or an interaction *Congruence*:*CT*, we calculated the difference of means of the Incongruent–Congruent conditions, and one sample t-tests for each CT level were performed. If the lateral repeated measures ANOVA demonstrated three-way interactions of CT-related factors with one of the topographic factors, the levels of the respective topographic factor were collapsed, e.g. the interaction *Laterality*:*Congruence*:*CT* lead to building left and right areas of interest by collapsing the levels of *Anteriority*.

### Results

#### Behavioral data

Overall, 79% of the memory probes were classified correctly (83% correct recognition, 75% correct rejection), suggesting that participants had processed the stimulus items attentively. Accuracy rates across the eight determiner-noun phrase conditions ranged between 74% (Sortal congruent) and 83% (Relational congruent). Accuracy rates for the adjective + noun phrases were 77% (correct), 79% (morphosyntactic violation) and 83% (semantic violation).

#### ERP data: Adjective-noun phrases

**Fig 2:**
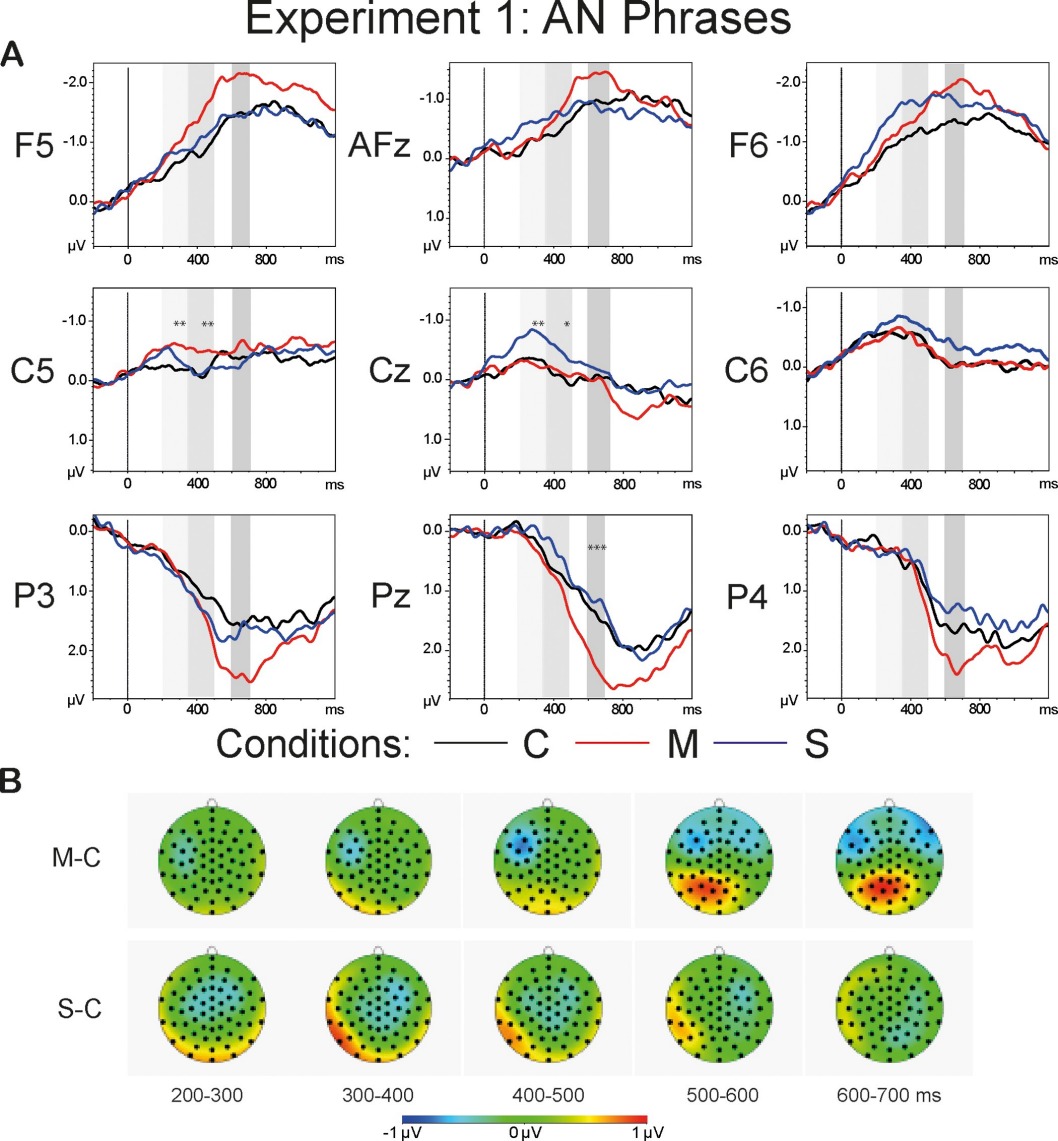
**Grand average waveforms (A) and the topographies of the difference waveforms of the type *Violation condition*–*Correct* (B).** The light-grey bars highlight the latency of 200–500 ms that comprises the time windows of the LAN and N400 components, i.e. 200–350 ms and 350–500 ms. The dark-grey bars highlight the P600 time window. The asterisks mark the results of one-way repeated measures ANOVAs within each ROI for each time window that reached significance (<0.001***, <0.01**, <0.05*). The topographies (B) depict the latency range of 200–700 ms in 100-ms steps: The N400 effect is observed in the *S-C* condition and it is most prominent at the central sites in the time window of 200–400 ms; the LAN effect can be detected in the *M-C* condition at the left temporal electrode sites, followed by the P600 effect at the posterior sites in the latency range of 600–700 ms.

Fig 2 shows the grand average waveforms of all AN conditions (A) and the topographies of the difference waveforms of the type *Violation condition*–*Correct* (B). The topographies are shown for the latency range of 200–700 ms in 100-ms steps. The light-grey bars highlight the latency of 200–500 ms that comprises the time windows of LAN and N400, i.e. 200–350 ms and 350–500 ms. The dark-grey bars highlight the P600 time window.

#### 0–200 ms

The repeated measures ANOVA for the lateral sites revealed neither any significant interactions with the factor *Condition* (*Anteriority*:*Laterality*:*Condition*: F(3.34, 76.88) = 1.22, p = 0.31) nor the main effect of *Condition*. The repeated measured ANOVA conducted for the midline sites, however, demonstrated significant main effects of *Anteriority* (F(1.41, 32.54) = 9.95, p = 0.001, η^2^ = 0.302) and *Condition* (F(1.99, 45.91) = 3.9, p<0.05, η^2^ = 0.145) but no significant interaction. Repeated measures one-way ANOVAs revealed a significant main effect of *Condition* at central electrode sites: F(2, 45.99) = 5.18, p<0.01, η^2^ = 0.184. Pairwise comparisons demonstrated a significant difference between *Correct* and *Semantic* violation conditions: t(23) = 3.01, p<0.05 (see Table 4 for an overview of the statistical analyses).

**Table 4:** Experiment 1, adjective-noun phrases: An overview of the statistical analyses.

Experiment 1	AL	AC	AR	CL	CC	CR	PL	PC	PR
**Repeated measures** **one-way** **ANOVA** ***F-values***									
**0–200 ms**					5.18**				
**200–350 ms**				5.92**	6.48**				
**350–500 ms**				6.77**	3.47*				
**600–700 ms**								15.8***	
**Paired t-tests** ***t-values***									
**0–200 ms**									
Correct vs. Morph									
Correct vs. Sem					3.01*				
**200–350 ms**									
Correct vs. Morph				3.13**					
Correct vs. Sem					2.95*				
**350–500 ms**									
Correct vs. Morph				2.76*					
Correct vs. Sem					2.55*				
**600–700 ms**									
Correct vs. Morph								-4.23***	
Correct vs. Sem									

The results are grouped according to the ROIs and types of analyses (ANOVA vs paired t-tests). Columns 2–10 represent ROIs: left anterior (AL), frontal (AC), right anterior (AR), left temporal (CL), central (CC), right temporal (CR), left posterior (PL), central-parietal (PC), and right posterior (PR). Rows 3–6 illustrate F-values for each ROI, whereas rows 8–11 show t-values that reached significance in the pairwise comparisons within the ROIs. A significant effect of *Condition* as tested by ANOVAs as well as significant differences between the *Correct* vs. *Morphosyntactic* violation, and the *Correct* vs. *Semantic* violation conditions as tested by post hoc pairwise comparisons are marked with asterisks (<0.001***, <0.01**, <0.05*).

#### 200–350 ms

The analysis of the AN conditions for the lateral sites revealed a significant three-way interaction of the type *Anteriority*:*Laterality*:*Condition*: F(2.99, 68.68) = 2.87, p<0.05, η^2^ = 0.111. The repeated measures ANOVA for the midline sites yielded no significant interaction but revealed significant main effects of *Anteriority* (F(1.6, 36.78) = 17.59, p<0.001, η^2^ = 0.433) and *Condition* (F(1.96, 45.09) = 6, p<0.01, η^2^ = 0.207). One-way repeated measures ANOVAs within each ROI yielded a significant effect of *Condition* at the left temporal (F(1.9, 43.8) = 5.92, p<0.01, η^2^ = 0.205) and central (F(1.95, 44.95) = 6.48, p<0.01, η^2^ = 0.220) electrode sites. The results of paired t-tests within the left temporal ROI revealed a significant difference between the *Correct* and the *Morphosyntactic* violation conditions: t(23) = 3.13, p = 0.01. Paired t-tests at central electrode sites demonstrated a significant difference between the *Correct* and the *Semantic* violation conditions: t(23) = 2.95, p<0.05.

#### 350–500 ms

The repeated measures ANOVA for the lateral sites revealed a significant three-way interactions of the type *Anteriority*:*Laterality*:*Condition*: F(2.7, 68.19) = 3.97, p<0.05, η^2^ = 0.147. The analysis for the midline sites yielded significant main effects of *Anteriority* (F(1.72, 39.61) = 31.88, p<0.001, η^2^ = 0.581) and *Condition* (F(1.88, 43.28) = 5.67, p<0.01, η^2^ = 0.198). One-way repeated measures ANOVAs within the ROIs revealed a significant effect of *Condition* at the left temporal (F(1.66, 38.21) = 6.77, p<0.01, η^2^ = 0.228) and central (F(1.89, 43.46) = 3.47, p<0.05, η^2^ = 0.131) electrode sites. The results of paired t-tests at the left temporal electrode sites yielded a significant difference between the *Correct* and the *Morphosyntactic* violation conditions: t(23) = 2.76, p<0.05. The comparison between the *Correct* and the *Semantic* violation conditions reached significance at the central sites: t(23) = 2.55, p<0.05.

#### 600–700 ms

**Fig 3:**
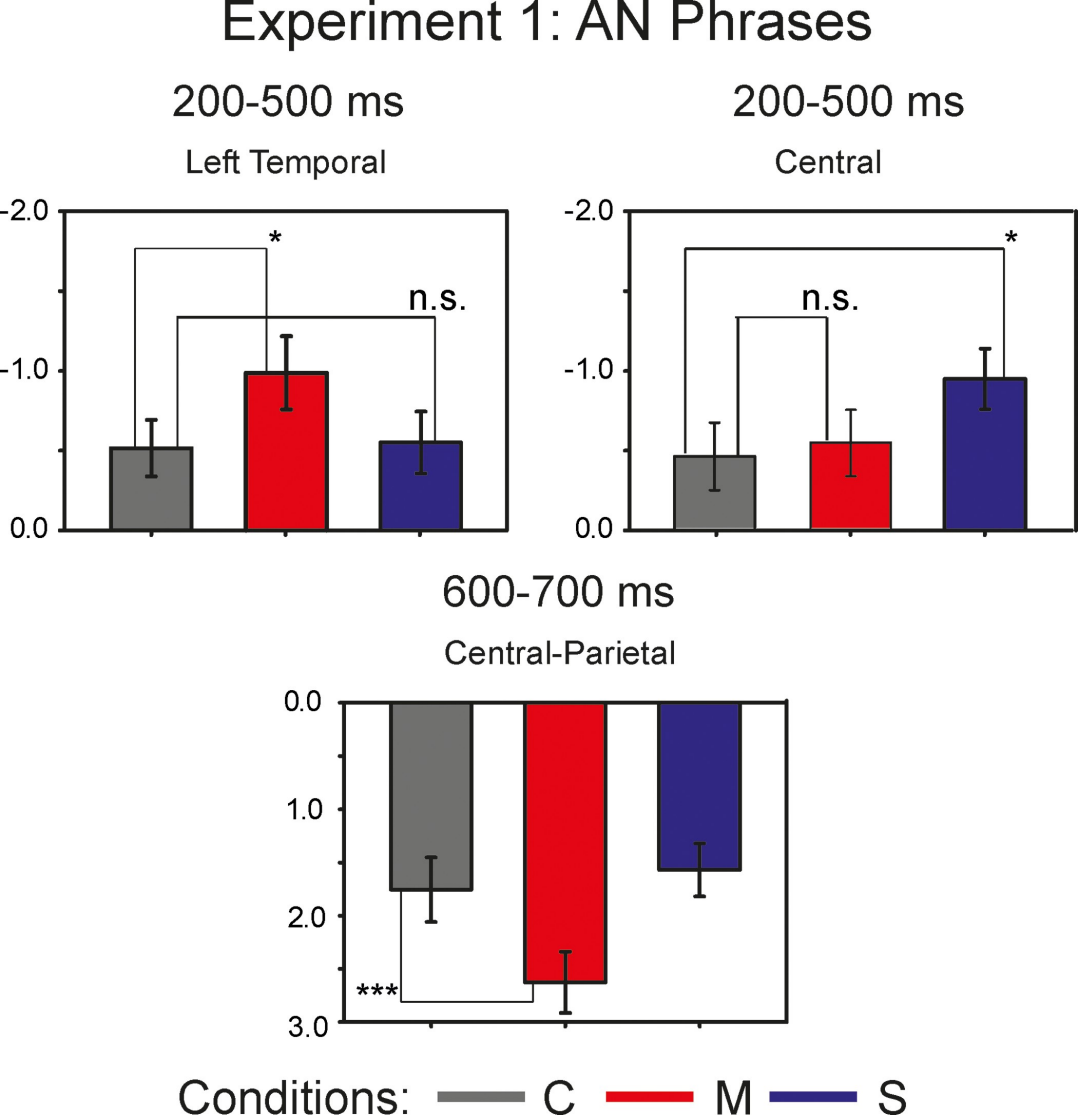
Bar Plots of the mean values of the AN conditions. The dark-grey bars represent the *Correct* condition, the red bars depict the *Morphosyntactic* violation condition, and the *Semantic* violation condition is marked with blue bars. The upper left part of the graph depicts the LAN in the latency of 200–500 ms at left temporal sites. The upper right part of the graph illustrates the N400 component at the central sites triggered by the *Semantic* violation condition. The lower part of the graph demonstrates the P600 component at the central-parietal electrode sites.

The repeated measures omnibus ANOVA for the lateral sites revealed significant two-way interactions of *Anteriority*:*Condition* (F(2.37, 54.58) = 5.72, p<0.01, η^2^ = 0.199) and *Laterality*:*Condition* (F(1.59, 36.53) = 5.04, p<0.05, η^2^ = 0.180), as well as a significant main effect of *Condition* (F(1.7, 39.04) = 4.94, p<0.05, η^2^ = 0.177). The repeated measures omnibus ANOVA for the midline sites demonstrated a significant two-way interaction *Anteriority*:*Condition* (F(2.34, 53.81) = 7.91, p<0.001, η^2^ = 0.256). For the further analyses, we collapsed *Anteriority* levels for the investigation of lateral effects, and *Laterality* levels for the investigation of Anteriority effects. The one-way repeated measures ANOVA reached significance at the left sites (F(1.91, 43.86) = 4.15, p<0.05, η^2^ = 0.153), and at the temporal sites (F(1.77, 40.71) = 4.46, p<0.05, η^2^ = 0.163). Pairwise comparisons yielded a significant difference between the *Correct* and the *Semantic* violation conditions at the temporal sites: t(23) = 2.98, p<0.05. The one-way repeated measures ANOVA conducted at the central-parietal sites revealed a main effect of *Condition* (F(1.88, 43.31) = 15.8, p<0.001, η^2^ = 0.407), with a post-hoc paired t-test showing a significant difference between the *Correct* and the *Morphosyntactic* violation conditions (t(23) = -4.23, p<0.001). Fig 3 illustrates the mean values for each AN condition within the ROIs with the largest effect size: the central sites for the N400 effect at 200–500 ms; the left temporal sites within the latency range of 200–500 ms for the LAN effect; the central-parietal sites for the P600 effect in the time window of 600–700 ms.

In summary, relative to the *Correct* condition, semantic violations demonstrated a negative deflection with a central distribution that reached significance in the time window of 0–200 and 200–500 ms. Morphosyntactic violations, compared to *Corrects*, showed a left lateralized negativity extending from 200 to 500 ms post recognition point. The left lateralized negativity was followed by a positivity at posterior electrode sites at 600–700 ms.

### ERP data: Concept types

#### 0–200 ms

**Fig 4:**
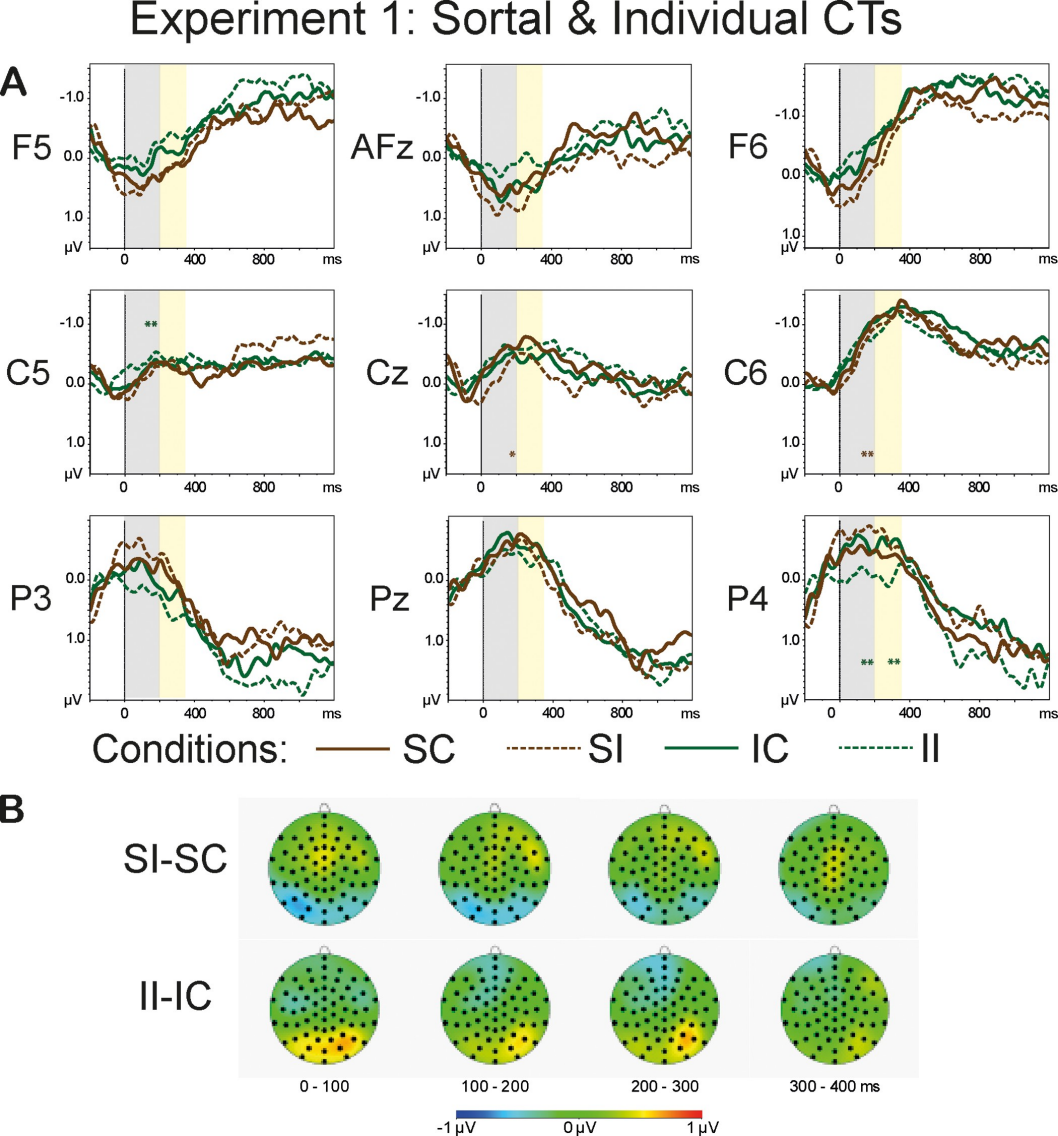
**Grand average waveforms of the Sortal and Individual CT conditions (A) and the topographies of the difference waveforms of the type *Incongruent*-*Congruent* (B).** The light-grey bars highlight the latency of 0–200 ms, the yellow bars highlight the time range of 200–350 ms that were used in the analyses. The solid lines depict the *Congruent* conditions; the dashed lines denote the *Incongruent* conditions. The CTs are color-coded: The *Sortal* CTs are marked brown, the *Individual* CTs are marked green. The asterisks denote the significance of the *Congruence* effect (<0.001***, <0.01**, <0.05*) within a given ROI: the brown asterisks correspond to the *Sortal* CT, the green asterisks mark the *Individual* CT. The topographies depict the latency range of 0–400 ms in 100-ms steps.

**Fig 5:**
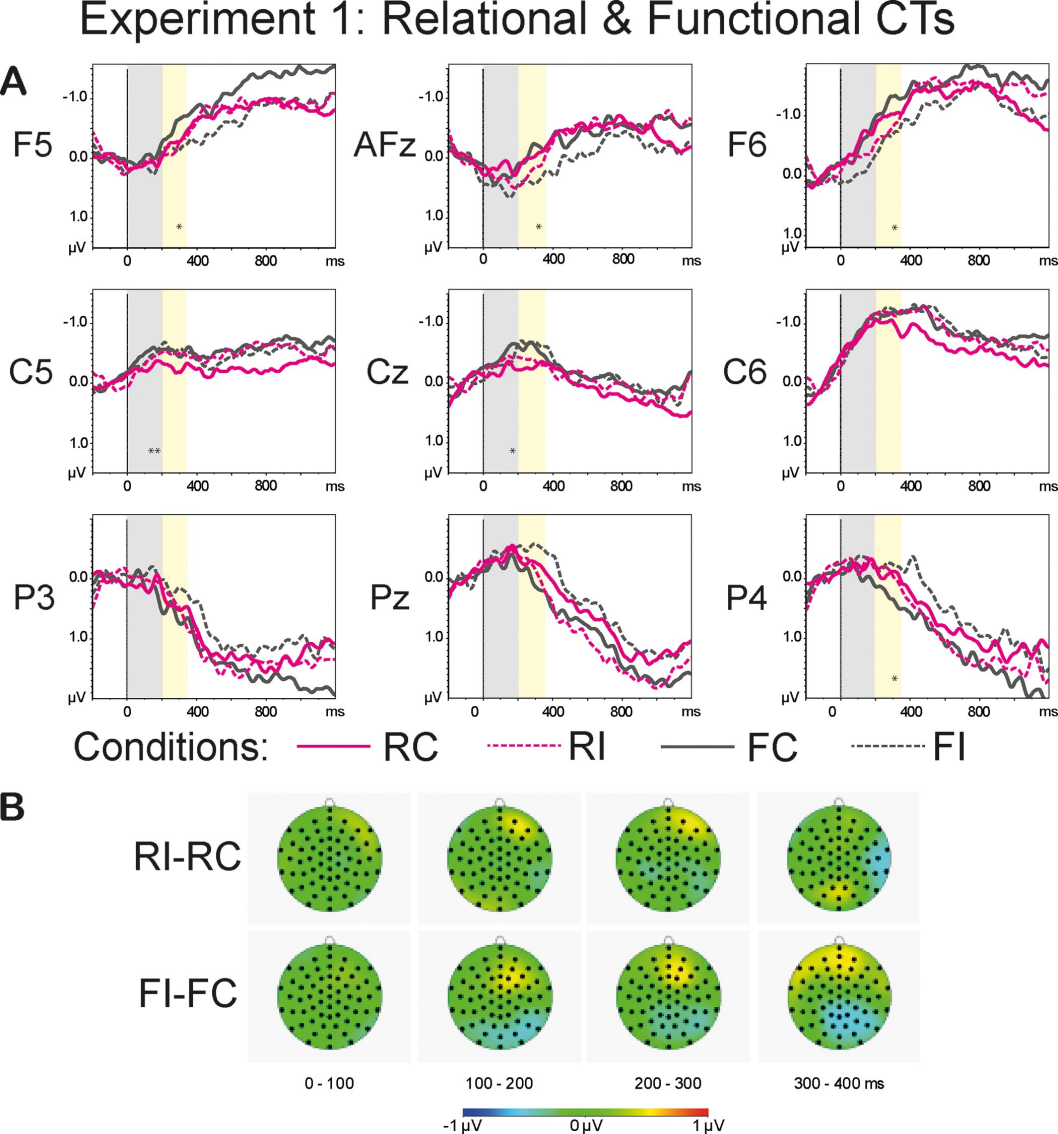
**Grand average waveforms of the Relational and Functional CT conditions (A) and the topographies of the difference waveforms of the type *Incongruent*-*Congruent* (B).** The light-grey bars highlight the latency of 0–200 ms, the yellow bars highlight the time range of 200–350 ms that were used in the analyses. The solid lines depict the *Congruent* conditions; the dashed lines denote the *Incongruent* conditions. The CTs are color-coded: The *Relational* CTs are marked pink, the *Functional* CTs are marked dark-grey. The asterisks denote the significance of the *Congruence* effect (<0.001***, <0.01**, <0.05*) within a given ROI: the pink asterisks correspond to the *Relational* CT, the dark-grey asterisks mark the *Functional* CT. The topographies depict the latency range of 0–400 ms in 100-ms steps.

The repeated measures omnibus ANOVA, for the lateral sites, revealed a significant four-way interaction of the type *Anteriority*:*Laterality*:*Congruence*:*CT* in the time range of 0–200 ms: F(3.97, 91.23) = 2.49, p<0.05, η^2^ = 0.098. The results also demonstrated a significant three-way interaction of the type *Anteriority*:*Congruence*:*CT* (F(3.39, 75.85) = 7.37, p<0.001, η^2^ = 0.243), a significant two-way interaction *Anteriority*:*CT* (F(2.7, 62.17) = 9.28, p<0.001, η^2^ = 0.288), and a significant main effect of *CT* (F(2.4, 55) = 8.28, p<0.001, η^2^ = 0.265). The results of the omnibus ANOVA for the midline sites also revealed a significant three-way interaction *Anteriority*:*Congruence*:*CT* (F(3.74, 68.07) = 5.51, p<0.001, η^2^ = 0.193 and a main effect of *CT* (F(2.87, 65.95) = 2.9, p<0.05, η^2^ = 0.112). Fig 4 shows grand averages of the *Sortal* (brown lines) and *Individual* (green lines) CT conditions (A) and the topographies of the difference waves of the type Incongruent-Congruent (B) in the range of 0–400 ms in 100-ms steps. Fig 5 demonstrates grand average waveforms of the *Relational* (pink lines) and *Functional* (dark-grey lines) CT conditions (A) and the topographies of the difference waveforms of the type Incongruent-Congruent (B) in the range of 0–400 ms in 100-ms steps.

The repeated measures ANOVAs within the ROIs revealed significant two-way interactions of the type *Congruence*:*CT* at the frontal (F(2.62, 60.26) = 5.88, p<0.01, η^2^ = 0.204), right anterior (F(2.69, 61.8) = 3.51, p<0.05, η^2^ = 0.132), left temporal (F(2.56, 59) = 3.99, p<0.05, η^2^ = 0.148), central (F(2.6, 59.76) = 4.4, p<0.05, η^2^ = 0.161), right temporal (F(2.59, 59.47) = 3.17, p<0.05, η^2^ = 0.121), and right parietal electrode sites (F(2.55, 58.75) = 7.44, p<0.001, η^2^ = 0.245). One-sample t-tests performed on the difference values demonstrated a significant *Congruence* effect for the *Individual* concepts at the left temporal (t(23) = -3.59, p<0.01) and right posterior (t(23) = 3.73, p<0.01) sites, and for the *Sortal* concepts in the central (t(23) = 2.76, p<0.05) and right temporal (t(23) = 3.56, p<0.01) ROIs.

#### 200–350 ms

The repeated measures omnibus ANOVA for the lateral sites failed to demonstrate a significant four-way interaction, however, we observed a significant three-way interaction of the type *Anteriority*:*Congruence*:*CT* (F(3.41, 78.52) = 4.67, p<0.01, η^2^ = o.169) and a two-way interaction *Anteriority*:*Congruence* (F(1.26, 28.99) = 4.19, p<0.05, η^2^ = 0.154). The analysis of the data with collapsed *Laterality* levels yielded a significant interaction *Congruence*:*CT* at the anterior lateral (F(2.71, 62.41) = 3.37, p<0.05, η^2^ = 0.128) and posterior lateral (F(2.56, 58.82) = 6.83, p<0.001, η^2^ = 0.229) sites. The repeated measures omnibus ANOVA for the midline sites demonstrated a significant three-way interaction *Anteriority*:*Congruence*:*CT* (F(3.6, 82.86) = 4.16, p<0.01, η^2^ = 0.153). Further two-way repeated measures ANOVAs within ROIs revealed a significant interaction *Congruence*:*CT* at the frontal sites: F(2.84, 65.32) = 5.5, p<0.05, η^2^ = 0.193. One-sample t-test yielded a significant effect of *Congruence* for the *Functional* concepts at the frontal (t(23) = 3.3, p<0.05), anterior lateral (t(23) = 3.07, p<0.05), and posterior lateral (t(23) = -3.33, p<0.05) sites, as well as for the *Individual* concepts at the posterior lateral sites (t(23) = 3.18, p<0.05). The overview of the statistical analyses is provided in Table 5.

**Table 5:** Experiment 1, Concept Type conditions: an overview of the statistical analyses.

Experiment 1	Anterior	Posterior	AC	CL	CC	CR	PR
**One-sample** **t-tests: 0–200 ms**							
**Individual**				-3.59**			3.73**
**Sortal**					2.76*	3.56**	
**Functional**				6.77**	3.47*		
**Relational**							
**One-sample** **t-tests: 200–350 ms**							
**Individual**		3.18*					
**Sortal**							
**Functional**	3.07*	-3.33*	3.3*				
**Relational**							

The results are grouped according to the ROIs and the type of analysis (one-sample t-tests). Columns 2–8 represent ROIs: anterior lateral (Anterior), posterior lateral (Posterior), frontal (AC), left temporal (CL), central (CC), right temporal (CR), and right posterior (PR). Rows 3–6 illustrate significant t-values for Concept types in the time window 0–200 ms post recognition point, whereas rows 8–11 show t-values that reached significance in the latency range of 200–350 ms. A significant effect of *Congruence* as tested by one-sample t-tests is marked with asterisks (<0.001***, <0.01**, <0.05*). Positive t-values indicate a positivity of the incongruent condition relative to the congruent condition, and vice versa.

#### 350–500 ms

The omnibus repeated measures ANOVA for the lateral sites demonstrated a significant three-way interaction *Anteriority*:*Laterality*:*Congruence* (F(1.97, 45.22) = 3.4, p<0.05, η^2^ = 0.129) and a two-way interaction *Anteriority*:*Congruence* (F(1.22, 28.06) = 4.97, p<0.05, η^2^ = 0.178). Further two-way repeated measures ANOVAs within the ROIs revealed a significant main effect of *Congruence* at the right anterior sites (F(1, 23) = 7.5, p<0.05, η^2^ = 0.246), and an interaction *Congruence*:*CT* (F(2.57, 59.16) = 3.29, p<0.05, η^2^ = 0.125) at the right posterior sites. The omnibus repeated measures ANOVA for the midline sites yielded a significant two-way interaction *Congruence*:*CT* (2.65, 60.97). The ANOVA with the collapsed Anteriority levels demonstrated a significant interaction *Congruence*:*CT*: F(2.63, 60.56) = 3.14, p<0.05, η^2^ = 0.120. One-sample t-test conducted with the difference values at the right anterior, right posterior, and midline sites failed to reach significance.

#### 600–700 ms

The omnibus repeated measures ANOVA for the lateral sites yielded no significant interactions with the factor *Congruence* (all p>0.05). However, we observed a significant interaction *Congruence*:*CT* at the midline sites: F(2.7, 62.21) = 3.22, p<0.05, η^2^ = 0.123.

### Discussion

Relative to the *Correct* condition, semantic incongruence between an adjective and a noun triggered a central negativity that started as early as 0–200 ms and became most prominent between 200 and 350 ms post recognition of the noun. The difference between the *Correct* and the *Semantic violation* conditions was compatible with the latency and the topographic distribution of the classic N400 effect [13, 14, 16, 17, 24, 70, 71, 80]. Gender agreement violation between an adjective and a noun in the AN conditions, relative to the *Correct* condition, elicited a left lateralized negativity that started at around 200 ms, lasted for about 400 ms, and was followed by a central-parietal positivity at around 600–700 ms post recognition point of the noun. This response pattern was consistent with the biphasic LAN-P600 pattern that has been reported in ERP studies investigating morphological number and gender agreement violations [53, 54, 64, 65, 75, 76, 79]. In sum, the results of the adjective-noun conditions show that our paradigm is sensitive to the classic ERP semantic and syntactic violation effects.

We argued that if incongruent determination affects the lexical retrieval of a noun or the semantic integration of the noun with the preceding determiner, it should be indexed by the size of the N400 effect. If, however, concept type shifts are supported by the same neuronal mechanisms that underlie morphosyntactic processing, incongruent determination should trigger LAN or/and P600 effects. The results of Experiment 1 support none of the two possibilities. The analyses of the determiner-noun conditions showed no overall *Congruence* effect, thus ruling out that concept type incongruence as such, irrespective of whether it is due to a uniqueness or relationality mismatch between determiner and noun, has similar processing consequences as semantic or syntactic violations. In contrast, we did observe concept type specific incongruence responses in the latency range of the N400 and the LAN effects. However, their topographic distributions and/or polarity differed from those of the classic effects. *Sortal* nouns preceded by incongruent compared to congruent determination elicited an ERP response with an N400-like topographic distribution but as a positive rather than a negative [13, 14, 16, 17, 24, 70, 71, 80] deflection. *Individual* nouns preceded by incongruent compared to congruent determination elicited a left temporal negativity and right posterior positivity in the 0–200 ms time window and a bilateral posterior positivity in the 200–350 ms time window. In the latter time window, *Functional* nouns preceded by incongruent compared to congruent determination elicited an anterior positivity and bilateral posterior negativity. Hence the timing and/or the distribution of these incongruence effects was incompatible with the LAN effect we observed for the gender-incongruent adjective-noun phrases in the 200–350 ms time window at left temporal sites [53, 54, 64, 65, 75, 76, 79]. In sum, Experiment 1 did not provide evidence that concept-type incongruences between determiners and nouns are perceived or processed similar to semantic or syntactic incongruences. Given that our control conditions using congruent and incongruent adjective-noun phrases yielded the expected ERP violation responses, we can rule out a lack of sensitivity as a possible reason for the lack of such responses to concept-type incongruent noun phrases. Note, however, that in order to keep the processing of the auditory stimuli as natural as possible and not to bias the participants towards semantic or grammatical processing [19, 28, 81–83], Experiment 1 did not employ an explicit linguistic task, such as a grammaticality or plausibility judgment, and the good performance on the memory probes only shows that the participants attended to and recollected the surface form of the presented noun phrases. the participants towards semantic or grammatical processing. We, therefore, cannot rule out that the processing of the determiner-noun phrases in the CT conditions may not have been sufficiently in-depth to elicit the classic ERP responses we observed for the adjective-noun phrases. In particular, the morphosyntactic ERP components have been reported to be task-sensitive and more likely to surface with explicit judgment tasks [19, 84]. We, therefore, decided to run a second study with a wellformedness judgment task, that would direct participants’ attention to the composition, but not expressly to the grammatical status or semantics of the experimental phrases.

## Experiment 2

The second experiment of the reported series of studies had the same experimental materials, EEG recording setup, and data analysis as the first study. However, the participants and the procedure were different. In the Methods section, we shall, therefore, describe only the issues that differed from those in the first experiment.

### Methods

#### Participants

Twenty-five right-handed (as assessed by the Edinburgh handedness test [68]) students of Heinrich-Heine-University, Düsseldorf (12 male, age range: 19–31 years, mean: 23.96, SD: 3.56) who were native speakers of Standard German took part in the study. One participant had to be excluded due to excessive muscular artifacts. The participants had normal or corrected-to-normal vision, reported no hearing impairments, no psychological and neurological disorders, and were not taking any psychoactive medication. The participants signed an informed consent form and were paid 16 € for participation.

#### Procedure

The participants were tested individually in a sound-attenuating booth. Their task was to listen to auditorily presented phrases and to perform a wellformedness judgment upon presentation of a visual cue. We did not specifically instruct the participants to pay attention to the grammatical structure or to the lexical status of the stimuli. Instead, we asked them to evaluate those phrases that they could easily use as ‘well-formed’ and phrases that could not be easily used or only used in specific contexts as ‘not well-formed’. The visual cue “Wohlgeformt?” (Well-formed?) was presented on the computer screen, and the wellformedness judgment had to be made about the auditorily presented phrase preceding the visual probe. The visual cues occurred after every 1–5 auditory phrases and were pseudo-randomized such that the wellformedness judgment had to made for each experimental condition 17 times throughout the experimental session. The participants were instructed to press the right mouse key if they considered the phrase well-formed, and to press the left mouse key if they considered the phrase NOT well-formed.

### Results

#### Behavioral data

Correct AN phrases were judged as well-formed in 89% of the cases. Noun phrases containing a morphosyntactic violation were accepted as well-formed in 6%, noun phrases containing a semantic violation in 37.5% of the cases. Concept-type congruent noun phrases were evaluated as well-formed in 86% (SC 92.3%, RC 87.7%, FC 82%, IC 82%) of the cases. Concept-type incongruent noun phrases were judged as well-formed in 82% (SI 93.5%, RI 90%, FI 87.7%, II 58.5%) of the cases.

#### ERP data: Adjective-noun phrases

**Fig 6:**
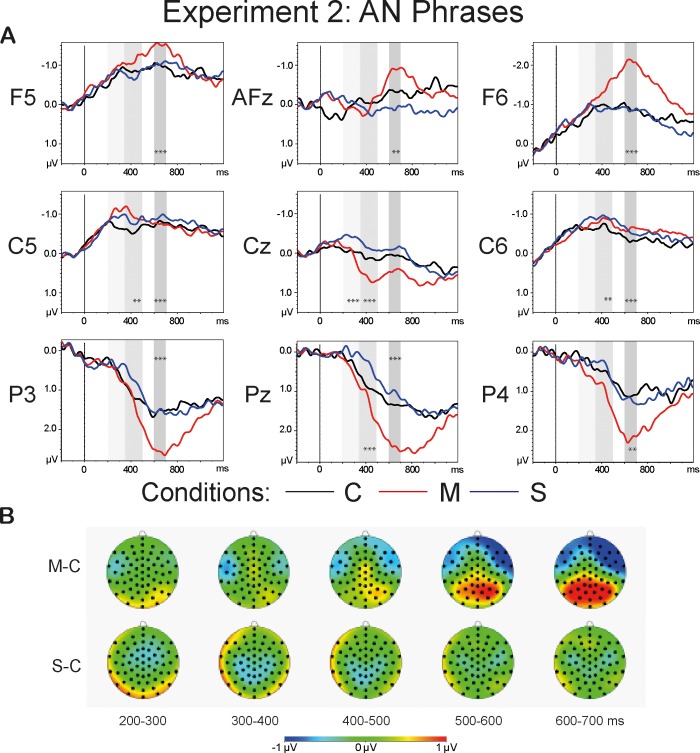
**Grand average waveforms (A) and the topographies of the difference waveforms of the type *Violation condition*–*Correct* (B).** The light-grey bars highlight the latency of 200–500 ms that comprises the time windows of the LAN and N400 components. The dark-grey bars highlight the P600 time window. The asterisks mark the results of one-way repeated measures ANOVAs within each ROI for each time window that reached significance (<0.001***, <0.01**, <0.05*). The topographies (B) depict the latency range of 200–700 ms in 100-ms steps: The N400 effect is observed in the *S-C* condition and it is most prominent at the central sites in the time window of 300–400 ms; the LAN effect can be detected in the *M-C* condition at the left temporal electrode sites, followed by the P600 effect at the posterior sites in the latency range of 600–700 ms.

Fig 6 demonstrates the grand average waveforms of the AN conditions (A) and the topographies of the difference waveforms of the type *Violation condition*–*Correct* (B). The topographies are shown for the latency range of 200–700 ms in 100-ms steps. The light-grey bars highlight the latency of 200–500 ms that entails the time windows of N400 (200–350 ms) and LAN (350–500 ms). The dark-grey bars highlight the P600 time window.

#### 0–200 ms

A repeated measures omnibus ANOVA conducted for lateral electrode sites yielded no main effect or interaction of the factor *Condition*. A repeated measures omnibus ANOVA for the midline sites, on the other hand, yielded significant main effects of *Anteriority* (F(1.18, 27.1) = 20.48, p<0.001, η^2^ = 0.471) and *Condition* (F(1.98, 45.66) = 6.11, p<0.01, η^2^ = 0.210). A one-way repeated measures ANOVA conducted with collapsed levels of *Anteriority* for the midline sites yielded a significant main effect of *Condition* (F(1.96, 45.05) = 4.94, p<0.05, η^2^ = 0.177) with both violation conditions being significantly more negative than the *Correct* condition (*Correct* vs. *Morphosyntactic violation*: t(23) = 2.79, p<0.05; *Correct* vs. *Semantic violation*: t(23) = 2.8, p<0.05).

#### 200–350 ms

A repeated measures omnibus ANOVA conducted for the lateral electrode sites yielded no main effect or interaction of the factor *Condition* (*Anteriority*:*Laterality*:*Condition*: F(3.25, 74.71) = 1.88, p = 0.14). The interaction with *Condition* also failed to reach significance for the midline sites. However, we observed significant main effects of *Anteriority* (F(1.36, 31.18) = 26.41, p<0.001, η^2^ = 0.535) and Condition (F(1.93, 44.29) = 8.38, p<0.001, η^2^ = 0.267). A repeated measures one-way ANOVA with collapsed levels of *Anteriority* for the midline sites yielded a significant main effect of *Condition*: F(1.94, 44.64) = 8.73, p<0.001, η^2^ = 0.275. Pairwise comparisons with collapsed levels of *Anteriority* for the midline sites revealed a significant difference between the *Correct* and the *Semantic* violation conditions: t(23) = 3.18, p<0.01 (see Table 6).

**Table 6:** Experiment 2, adjective-noun phrases: An overview of the statistical analyses.

Experiment 2	Anterior	Temporal	Posterior	Midline	AC	PC
**Repeated measures one-way ANOVA** ***F-values***						
**0–200 ms**				4.94*		
**200–350 ms**				8.73***		
**350–500 ms**		8.37**		10.84***		
**600–700 ms**	13.7***	11.23***	17.42***		6.42**	16.63***
**Paired t-tests** ***t-values***						
**0–200 ms**						
Correct vs. Morph				2.79*		
Correct vs. Sem				2.8*		
**200–350 ms**						
Correct vs. Morph						
Correct vs. Sem				3.18**		
**350–500 ms**						
Correct vs. Morph		3.72**		-3.16**		
Correct vs. Sem						
**600–700 ms**						
Correct vs. Morph	4.47***	4.4***	-4.76***		2.86*	-4.34***
Correct vs. Sem		2.68*				

The results are grouped according to the ROIs and types of analyses (ANOVA vs paired t-tests). Columns 2–7 represent ROIs: Anterior, Temporal, and Posterior that were built by collapsing *Laterality* levels (left and right), Midline ROI that was calculated for the midline electrodes by collapsing the levels of *Anteriority*, frontal (AC), and central-parietal (PC). Rows 3–6 illustrate F-values for each ROI, whereas rows 8–11 show t-values that reached significance in the pairwise comparisons within the ROIs. A significant effect of *Condition* as tested by ANOVAs as well as significant differences between the *Correct* vs. *Morphosyntactic* violation, and the *Correct* vs. *Semantic* violation conditions as tested by post hoc pairwise comparisons are marked with asterisks (<0.001***, <0.01**, <0.05*).

#### 350–500 ms

A repeated measures omnibus ANOVA conducted for the lateral sites revealed a significant main effect of *Condition* (F(1.67, 38.38) = 5.32, p<0.05, η^2^ = 0.188) and a two-way interaction *Anteriority*:*Condition* (F(2.37, 54.59) = 3.59, p<0.05, η^2^ = 0.135). The analysis of variance at the midline sites yielded significant main effects of *Anteriority* (F(1.24, 28.61) = 32.09, p<0.001, η^2^ = 0.582) and *Condition* (F(1.88, 43.16) = 9.96, p<0.001, η^2^ = 0.302). A repeated measures two-way ANOVA with collapsed levels of *Laterality* revealed a significant interaction *Anteriority*:*Condition* (F(2.37, 54.59) = 3.59, p<0.05, η^2^ = 0.135. One-way repeated measures ANOVAs conducted for the different levels of *Anteriority* yielded a significant main effect of *Condition* at the temporal sites (F(1.66, 38.17) = 8.37, p<0.01, η^2^ = 0.267). Pairwise t-tests at the temporal electrode sites demonstrated a significant difference between the *Correct* and the *Morphosyntactic* violationconditions: t(23) = 3.72, p<0.01 (see Table 6). A one-way repeated measures ANOVA conducted with collapsed levels of *Anteriority* for the midline sites revealed a significant main effect of *Condition* (F(1.84, 42.38) = 10.84, p<0.001, η^2^ = 0.320) with a significant difference between the *Correct* and the *Morphosyntactic* violation conditions (t(23) = -3.16, p<0.01).

#### 600–700 ms

**Fig 7:**
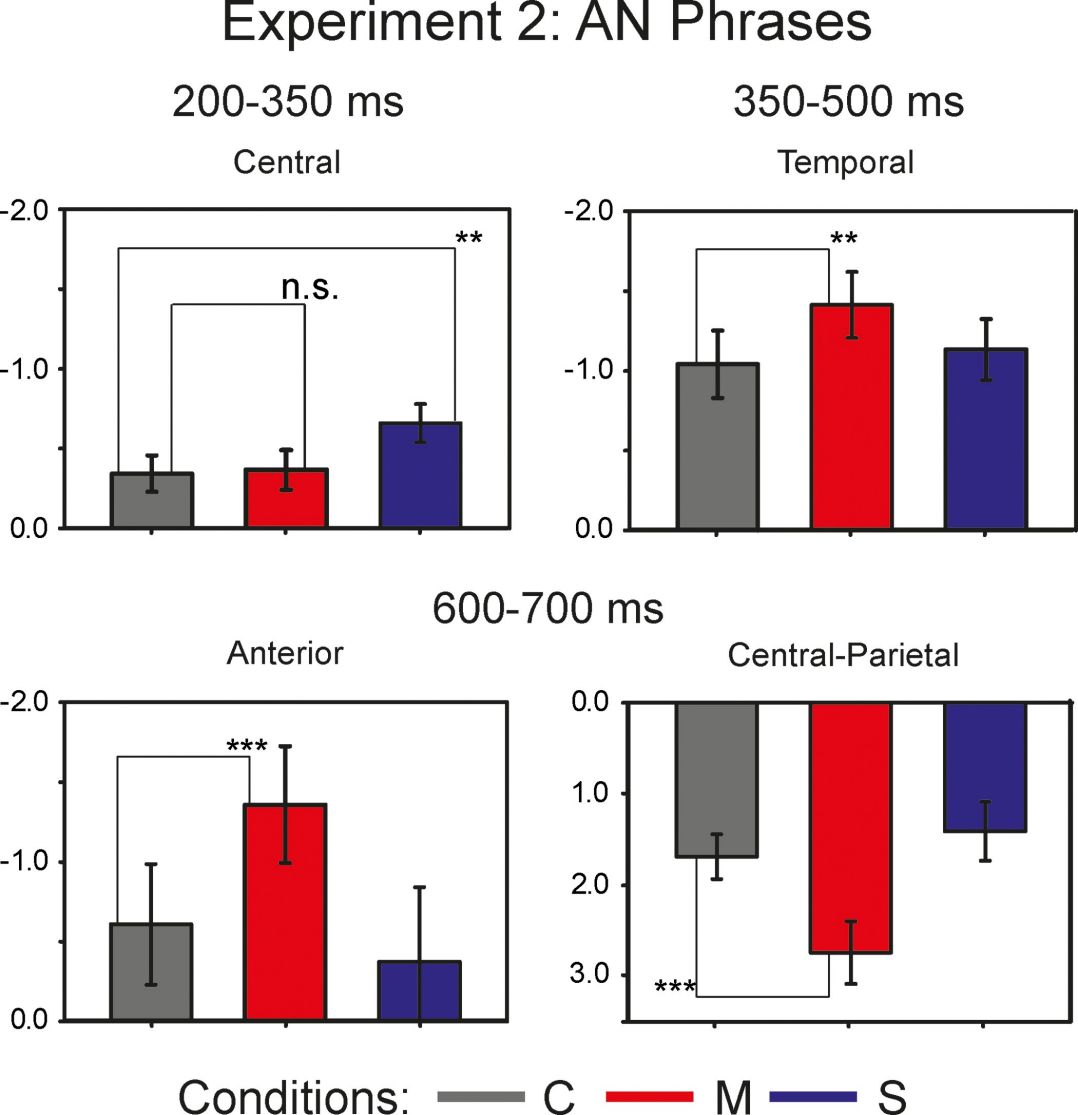
Bar Plots of the mean values of the AN conditions. The dark-grey bars represent the *Correct* condition, the red bars depict the *Morphosyntactic* violation condition, and the *Semantic* violation condition is marked with blue bars. The upper left part of the graph illustrates the N400 component at the central sites triggered by the *Semantic* violation condition. The upper right part of the graph depicts the LAN in the latency of 300–500 ms at left temporal sites. The lower part of the graph demonstrates the sustained negativity at the anterior sites and the P600 component at the central-parietal electrode sites.

A repeated measures omnibus ANOVA conducted for the lateral sites demonstrated a significant main effect of *Condition* (F(1.62, 37.18) = 7.21, p<0.01, η^2^ = 0.239) and a two-way interaction *Anteriority*:*Condition* (F(2.34, 53.93) = 15.58, p<0.001, η^2^ = 0.404). A repeated measures omnibus ANOVA for the midline sites revealed a significant interaction *Anteriority*:*Condition* (F(2.31, 53.25) = 12.68, p<0.001, η^2^ = 0.355). One-way repeated measures ANOVAs conducted with collapsed levels of *Laterality* yielded a significant main effect of *Condition* at anterior (F(1.82, 41.95) = 13.7, p<0.001, η^2^ = 0.373), temporal (F(1.83, 42.21) = 11.23, p<0.001, η^2^ = 0.328), and posterior (F(1.75, 40.18) = 17.42, p<0.001, η^2^ = 0.431) electrode sites. Pairwise comparisons between the *Correct* and the *Morphosyntactic* violation conditions reached significance at anterior (t(23) = 4.47, p<0.001), temporal (t(23) = 4.4, p<0.001), and posterior (t(23) = -4.76, p<0.001) sites. The comparison between the *Correct* and the *Semantic* violation conditions was significant only at temporal sites (t(23) = 2.68, p<0.05). One-way repeated measures ANOVAs for the midline ROIs reached significance for the frontal (F(1.84, 42.4) = 6.42, p<0.01, η^2^ = 0.218), and central-parietal (F(1.96, 45) = 16.63, p<0.001, η^2^ = 0.420) electrode sites. Post hoc paired t-tests yielded a significant difference between the *Correct* and the *Morphosyntactic* violation conditions for the frontal (t(23) = 2.86, p<0.05) and central-parietal sites (t(23) = -4.34, p<0.001). Fig 7 illustrates the mean values for each AN condition within the ROIs with the largest effect size: the central sites for the N400 effect at 200–350 ms; the temporal sites within the latency range of 350–500 ms for the lateralized negativity; the anterior sites for sustained negativity and central-parietal sites for the P600 effect in the time window of 600–700 ms.

### ERP data: Concept types

#### 0–200 ms

**Fig 8:**
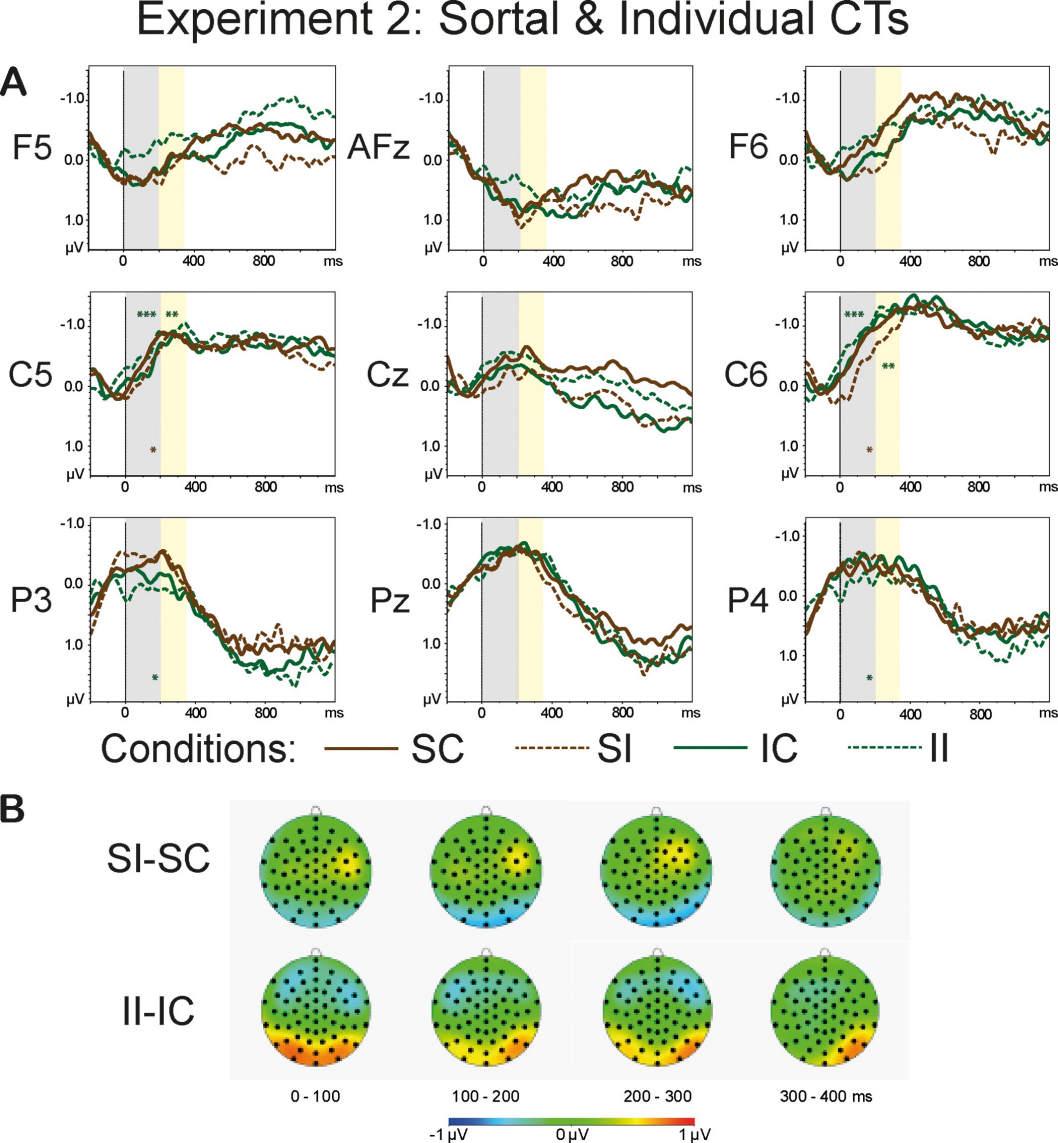
**Grand average waveforms of the Sortal and Individual CT conditions (A) and the topographies of the difference waveforms of the type *Incongruent*-*Congruent* (B).** The light-grey bars highlight the latency of 0–200 ms, the yellow bars highlight the time range of 200–350 ms that were used in the analyses. The solid lines depict the *Congruent* conditions; the dashed lines denote the *Incongruent* conditions. The CTs are color-coded: The *Sortal* CTs are marked brown, the *Individual* CTs are marked green. The asterisks denote the significance of the *Congruence* effect (<0.001***, <0.01**, <0.05*) within a given ROI: the brown asterisks correspond to the *Sortal* CT, the green asterisks mark the *Individual* CT. The topographies depict the latency range of 0–400 ms in 100-ms steps.

**Fig 9:**
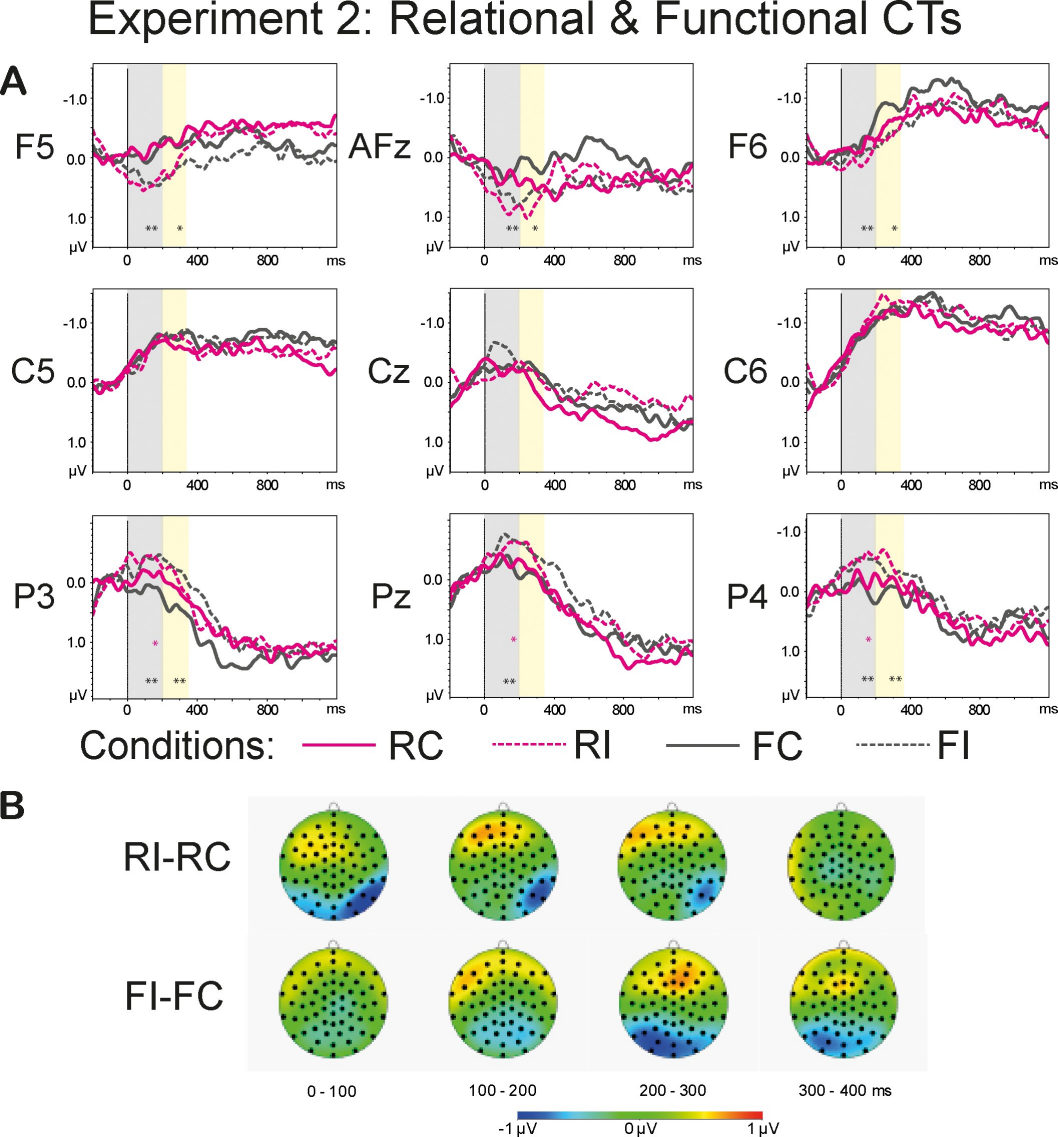
**Grand average waveforms of the Relational and Functional CT conditions (A) and the topographies of the difference waveforms of the type *Incongruent*-*Congruent* (B).** The light-grey bars highlight the latency of 0–200 ms, the yellow bars highlight the time range of 200–350 ms that were used in the analyses. The solid lines depict the *Congruent* conditions; the dashed lines denote the *Incongruent* conditions. The CTs are color-coded: The *Relational* CTs are marked pink, the *Functional* CTs are marked dark-grey. The asterisks denote the significance of the *Congruence* effect (<0.001***, <0.01**, <0.05*) within a given ROI: the pink asterisks correspond to the *Relational* CT, the dark-grey asterisks mark the *Functional* CT. The topographies depict the latency range of 0–400 ms in 100-ms steps.

Fig 8 shows grand averages of the *Sortal* (brown lines) and *Individual* (green lines) CT conditions (A) and the topographies of the difference waves (incongruent-congruent) (B) in the range of 0–400 ms in 100-ms steps. Fig 9 demonstrates grand average waveforms of the *Relational* (pink lines) and *Functional* (dark-grey lines) CT conditions (A) and the topographies of the difference waveforms of the type Incongruent-Congruent (B) in the range of 0–400 ms in 100-ms steps. Arepeated measures omnibus ANOVA conducted for the lateral sites revealed a significant interaction *Anteriority*:*Congruence*:*CT* (F(2.6, 59.58) = 7.41, p<0.001, η^2^ = 0.244). This interaction also reached significance for the midline sites: F(2.77, 63.79) = 4.25, p<0.01, η^2^ = 0.156. Two-way repeated measures ANOVAs conducted for the different levels of *Anteriority* with collapsed levels of *Laterality* (left and right) yielded a significant interaction *Congruence*:*CT* for anterior (F(2.14, 49.21) = 6.32, p<0.01, η^2^ = 0.216), temporal (F(2.36, 54.36) = 13.58, p<0.001, η^2^ = 0.371), and posterior (F(2.27, 52.33) = 7.54, p<0.001, η^2^ = 0.247) sites. Two-way repeated measures ANOVAs conducted for the midline sites also revealed a significant interaction *Congruence*:*CT* for the frontal (F(2.35, 53.99) = 3.76, p<0.05, η^2^ = 0.140), central (F(2.42, 55.6) = 5.06, p<0.01, η^2^ = 0.180), and central-parietal (F(2.57, 59.15) = 3.38, p<0.05, η^2^ = 0.128) sites. One-sample t-tests demonstrated a significant *Congruence* effect triggered by the shift of the *Individual* CT at temporal (t(23) = -4.87, p<0.001) and posterior (t(23) = 2.96, p<0.05) regions. The difference between the congruent and incongruent *Functional* CT reached significance for the central-parietal (t(23) = -3.11, p<0.05), anterior (t(23) = 3.99, p<0.01) and (t(23) = -3.59, p<0.01) posterior sites. There was also a significant effect of *Congruence* for *Sortal* CT at temporal sites (t(23) = 3.26, p<0.05) and *Relational* CT at posterior sites (t(23) = -2.81, p<0.05). The overview of the statistical analyses is provided in Table 7.

**Table 7:** Experiment 2, concept type conditions: An overview of the statistical analyses.

Experiment 2	Anterior	Temporal	Posterior	PC
**One-sample** **t-tests: 0–200 ms**				
**Individual**		-4.87***	2.96*	
**Sortal**		3.26*		
**Functional**	3.99**		-3.59**	-3.11*
**Relational**			-2.81*	
**One-sample** **t-tests: 200–350 ms**				
**Individual**		-3.6**		
**Sortal**				
**Functional**	3.37*		-4.03**	
**Relational**				

The results are grouped according to the ROIs and the type of analysis (one-sample t-tests). Columns 2–8 represent ROIs: lateral Anterior, Temporal, and Posterior, and the central-parietal ROI (PC). Rows 3–6 illustrate significant t-values for Concept types in the time window 0–200 ms post recognition point, whereas rows 8–11 show t-values that reached significance in the latency range of 200–350 ms. A significant effect of *Congruence* as tested by one-sample t-tests is marked with asterisks (<0.001***, <0.01**, <0.05*). Positive t-values indicate a positivity of the incongruent condition relative to the congruent condition, and vice versa.

#### 200–350 ms

An omnibus repeated measures ANOVAs conducted for the lateral and midline sites revealed a significant three-way interaction *Anteriority*:*Congruence*:*CT* (F(3.2, 70.44) = 4.17, p<0.01, η^2^ = 0.159 and F(3.08, 67.82) = 4.12, p<0.01, η^2^ = 0.158, respectively). Two-way repeated measures ANOVAs conducted for the different levels of *Anteriority* with collapsed levels of *Laterality* (left and right) yielded a significant interaction *Congruence*:*CT* for anterior (F(2.38, 52.48) = 3.64, p<0.05, η^2^ = 0.142), temporal (F(2.68, 61.62) = 4.17, p<0.05, η^2^ = 0.153), and posterior (F(2.7, 62.14) = 5.39, p<0.01, η^2^ = 0.190) regions. Two-way repeated measures ANOVAs for the midline sites failed to reveal any significant main effects or interactions. One-sample t-tests yielded a significant *Congruence* effect triggered by the incongruent *Individual* CT at temporal sites (t(23) = -3.6, p<0.01). The effect of *Congruence* for the *Functional* CT reached significance for anterior (t(23) = 3.37, p<0.05) and posterior (t(23) = -4.03, p<0.01) lateral sites.

#### 350–500 ms

Repeated measures omnibus ANOVAs conducted for the lateral and midline sites revealed no significant main effects or interactions.

#### 600–700 ms

A repeated measures omnibus ANOVA conducted for the lateral electrode sites revealed a significant two-way interaction *Anteriority*:*Congruence* (F(1.45, 33.3) = 4.43, p<0.05, η^2^ = 0.161). One-way repeated measures ANOVAs conducted for the different levels of *Anteriority* with collapsed levels of *Laterality* and *CT* yielded a significant main effect of *Congruence* for the frontal sites (F(1, 23) = 4.77, p<0.05, η^2^ = 0.172), with the mean value of congruent conditions being more negative than that of incongruent conditions.

### Discussion

In our second experiment we employed a wellformedness judgment task to encourage a more in-depth processing of the auditorily presented noun phrases. The participants’ judgments show different degrees of acceptability between morphosyntactic agreement violations and semantic incongruences on the one hand and concept type incongruences on the other. Noun phrases containing gender agreement violations (*interessantes Artikel*, interesting (n.) article (m.)) were downright rejected and noun phrases containing semantic incongruences (*koffeinfreier Artikel*, decaffeinated article) were only accepted as possibly usable in about one third of the cases. In contrast, most noun phrases containing concept type incongruences (*der Stein*, the stone; *das Ohr*, the ear; *eine Mutter*, a mother) were as well accepted as their congruent counterparts (*ein Stein*, *sein Ohr*, *seine Mutter)*. Only incongruent noun phrases containing *individual* nouns (*sein Papst*, his Pope) were judged as somewhat less freely usable. The participants’ judgements thus correspond to the corpus-based co-occurrence frequencies reported in Table 2, that also were of the same order of magnitude for congruent and incongruent noun phrases, except for *individual* nouns with incongruent determination. The judgement data, furthermore, suggest that neither the longer lexical decision latencies observed for CT-incongruent noun phrases in behavioral experiments [2, 3] are not likely to be due to some perceived ‘oddity’ of these phrases. To the contrary, noun phrases with incongruent determination are perceived as normal, corresponding to the observation from corpus-analyses [2] that incongruent determination is in fact quite frequent. In other words, in conjunction with corpus data, our participants’ judgments show that in everyday communication concept type shifts are pervasive and not perceived as incongruences.

The electrophysiological data of Experiment 2 largely replicated those of Experiment 1. The topographic distribution and the latency of the effect of the *Semantic violation* condition were slightly different from those reported in the first experiment. Nevertheless, the observed effect was again compatible with the latency and the topographic distribution of the classic N400 effect [13, 14, 16, 17, 24, 70, 71, 85]. Likewise, gender agreement violations between an adjective and a noun in the AN conditions triggered a response pattern that was consistent with the classic biphasic LAN-P600 pattern observed in ERP studies investigating number and gender agreement violations [53, 54, 64, 65, 75, 76, 79]. However, unlike in Experiment 1, the *Morphosyntactic violation* condition elicited an additional late anterior negativity, that is compatible with a sustained negativity that has been reported to reflect second-pass syntactic processing [86–89]. Somewhat speculatively one might assume that this additional sustained negativity may be due to more in-depth morphosyntactic processing as a result of the judgment task.

The analyses of the determiner-noun conditions again showed no overall *Congruence* effect and at least in part similar concept-type specific incongruence effects as in Experiment 1. We found a *Congruence* effect for noun phrases with *Individual* nouns at temporal and posterior lateral sites in the time window of 0–200 ms. This effect was similar in anteriority to that reported in Experiment 1, however, in Experiment 2 it had a bilateral distribution: a temporal negativity and a posterior positivity. In the time window of 200–350 ms, the incongruent *Individual* condition evoked a bilateral temporal negativity. The effect elicited by incongruent noun phrases with *Sortal* nouns was observed only in the early time window as a bilateral temporal positivity. We also replicated the results of Experiment 1 with respect to the incongruence response for *Functional* nouns: an anterior positivity and a bilateral posterior negativity between 200–350. Moreover, this effect was already significant in the early time window. Unlike in Experiment 1, there was a significant incongruence response for *Relational* nouns in the form of a bilateral posterior negativity in the time range of 0–200 ms post recognition point.

In sum, with respect to the classic ERP effects, the results of Experiment 2 largely replicated those of Experiment 1, thus excluding the possibility that the lack of classic ERP responses to concept-type incongruences in the previous experiment was due to a relatively shallow processing of the presented determiner-noun phrases in the absence of a linguistic task.

## General discussion

The Theory of Concept Types and Determination [1] postulates four concept types (Sortal, Individual, Relational, and Functional) that are characterized by specific combinations of uniqueness and relationality features. For each concept type there is a default determination that is congruent with respect to the uniqueness and relationality features it requires. The combination of nouns with incongruent determination leads to a concept type shift: *his father* (functional concept: unique, relational)–*a father* (sortal concept).

The objective of the present study was to establish the neural correlates of concept type incongruences. We argued that if incongruent determination affects the lexical retrieval of the noun or the semantic integration of the noun with the preceding determination, this should be reflected in the magnitude of the N400 effect. If, on the other hand, the mode of determination affects the morphosyntactic processing of a concept type, incongruent determination should trigger LAN or/and P600 effects. To investigate the electrophysiological signature of concept type incongruences, we conducted two ERP experiments with identical stimulus materials. In Experiment 1, participants simply listened to the stimuli. In Experiment 2, they performed a wellformedness judgment task. The stimuli employed in the experiments included eight concept type conditions (congruent and incongruent for each of the four CTs) and three adjective + noun conditions: correct, a semantic violation, and a morphosyntactic, i.e. gender, violation. The adjective+noun conditions served as a baseline that would trigger the classic linguistic ERP components: N400, LAN, and/or P600. Our goal was to compare the effects elicited by concept type incongruences to the violation effects elicited in the adjective + noun conditions.

### Classic ERP effects

The ERP results of both experiments revealed a comparable response pattern for the adjective + noun phrases. The semantic violation condition triggered a central (Experiment 1&2) and central-parietal (Experiment 2) negativity in the time window of about 200–500 ms, relative to the Correct condition, which corresponds to the latency and topography of the N400 effect [13, 28, 70, 71]. The gender violation condition elicited a left temporal negativity in the time range of 200–500 ms in Experiment 1 and in the latency of 350–500 ms in Experiment 2, consistent with the LAN effect [40, 54, 75, 76, 79]. In both experiments, the left temporal negativity was followed by a sustained anterior negativity and a central-parietal/ posterior positivity compatible with the P600 effect [54, 65]. Thus, independent of the presence of a judgment task, our experimental paradigm was able to detect the classic ERP effects.

The results of the concept type analyses demonstrated that (i) there was no general incongruence response across the four concept types and that (ii) there were no overlapping patterns of the brain responses to incongruent determiner-noun phrases of the different concept types and the classic responses to semantic and morphosyntactic violations in the canonical latencies, i.e. 200–350, 350–500, and 600–700 ms. Both results were obtained irrespective of whether participants simply listened to noun phrases or were actively engaged in a judgment task, suggesting that they are robust and cannot be explained by too shallow processing of the stimuli.

Based on the first result we must conclude that there is no electrophysiological evidence for the processing of concept type congruence per se nor for a general type shifting process. By contrast, our finding of different incongruence responses for the four concept types is compatible with distinct congruence detection or shifting processes for uniqueness and relationality features. However, our second result suggests that also such distinct processes are not supported by the same neural mechanisms that underlie lexical retrieval and syntactic processing. That concept type incongruence does not seem to elicit an enhanced N400 as an index of the relative difficulty of lexical retrieval, which is in line with the behavioral data on the concept type incongruence effect reported by Brenner and colleagues [2, 3]. These authors also concluded that the CT-congruence effect found in lexical decision experiments must arise post-lexically because it disappeared in a phoneme monitoring task that selectively taps into lexical retrieval.

The absence of the classic morphosyntactic violation effects for concept type incongruences suggests that the binary uniqueness and relationality features assumed in Löbner’s (1) theory may be lexically specified but do not seem to have the same status as lexically specified syntactic gender features for which a mismatch triggers robust violations effects.

### Novel ERP effects

Given that the concept-type specific ERP response patterns we observed were unlike the classic ERP violation responses, the question arises what underlying processes might instead have driven these responses. For the Sortal concept type incongruence elicited a central/temporal positivity. For the Individual concept type incongruence triggered a central lateralized negativity (both studies) and a left (Exp.1) or bilateral (Exp.2) posterior positivity. For the Relational concept type incongruence surfaced as an anterior positivity only in Experiment 2. The response pattern triggered by the Functional concept type with incongruent determination was similar in both experiments: an anterior positivity and a posterior negativity. The direction and the extent of the electrophysiological responses to incongruences thus depended on the concept type: whereas the incongruences in Sortal, Relational, and Functional concept types elicited an anterior or central positivity and a posterior negativity, the shift of the Individual concept type triggered a temporal negativity and a posterior positivity.

As discussed above, the *Congruence* effects observed in the different concept type conditions do not seem to be elicited by difficulties in semantic and/or morphosyntactic processing. The reported effect must have been driven by a qualitatively distinct mechanism. A large number of recent studies suggest that the perception of language does not rely on a simple syntactic parser that combines meanings of separate morphemes or words into larger units according to existing morphosyntactic rules [90–95]. Instead, language processing makes use of several mechanisms: the aforementioned classic parser, a possible independent semantic parser ([90, 95–98] but see [99] for a different account), world knowledge [100–103], and sentence/discourse context [18, 104–107]. The existence of the independent semantic parser was successfully tested in the studies on enriched composition, many of them investigating complement coercion [95, 97, 98, 108–113]. Complement coercion occurs when a complement NP of a verb has to be shifted from an entity to an event: e.g. *John began* ***the book*** [10]. The semantic requirement of the verb “to begin” is that it should be combined with a complement of the semantic type *event*: e.g. *John began* ***the fight***. Although the former example demonstrates a type mismatch, every speaker of the given language will interpret this example as “*John began doing something related to the book*”. Therefore, the surface structure of the sentence remains unchanged, and the type-shifting operation occurs at the level of semantics. The results of the psycho- and neurolinguistic experiments revealed that the type-shifting operation involved significant processing costs [94, 97, 98, 108, 112, 113]. An MEG study on coercion and compositionality by Pylkkänen and McElree [97] recorded event-related fields (ERFs) generated by semantically anomalous nouns, coerced nouns, and control sentences. Coercion failed to modulate activity in the areas related to semantic or syntactic processing such as the left temporal lobe structures, or the left inferior frontal gyrus. Instead, the authors observed an effect that they named the *anteriormidline field* (AMF) generated by a midline source in the ventromedial prefrontal cortex (vmPFC). The AMF was not modulated by semantic anomalies and could thus be dissociated from the lexico-semantic processing that is supported by the left temporal structures [114–118]. More recent MEG studies showed that the AMF is also sensitive to aspectual coercion [96] and that vmPFC is involved in the perception and production of adjective + noun phrases [119, 120].

An ERP study by Baggio and colleagues [94] delivered further evidence for the increased processing costs of coercing sentences relative to the neutral ones. However, the authors could not establish a coercion-specific ERP effect, as the coerced nouns triggered a negative-going shift at central sites that, though long-lasting, could not be entirely dissociated from the N400 component. On the other hand, as the N400 reflects difficulties in the integration of the word meaning [13, 121–123], the sustained negativity observed by Baggio and colleagues could indeed be the N400 effect reflecting the costs of integration of the coerced noun into the sentence context. In contrast to Baggio’s study, the shifted nouns in our experiments failed to elicit an ERP effect compatible with any classic ERP component. The only condition that triggered an anterior and, in part, central negativity was the incongruent Individual condition. The topographic distribution of this effect however did not match the effect reported by Baggio and colleagues [94].

The incongruent concept type conditions in our study were implemented by shifting one or both of the features Uniqueness [U] and Relationality [R]. The incongruent *Individual* condition was created by shifting the feature Relationality from [-R] to [+R]. Interestingly, relational concepts with congruent possessive determination–*his ear*, *his mother*–triggered a similar response pattern as the incongruent Individual concepts (*his pope*), i.e. anterior/temporal negativity and a posterior positivity, raising the possibility that this response pattern might be attributed to the pragmatic processing of the possessive determination. A preceding possessive determiner might trigger the assessment of the subsequent noun as a potential filler for the argument slot.

A *congruence* effect that did not involve shifting of the feature Relationality was observed for the Sortal concept type: the shift of Uniqueness [-U]→[+U] resulted in a temporal positivity and a bilateral posterior negativity. Interestingly, the *Sortal* concept (*a stone*) and the *Individual* concept (*the pope*) conditions had a similar morphology of the grand average ERP waveforms, the incongruent *Sortal* condition (*the stone*) being more positive at anterior and central electrodes than the congruent *Sortal* and *Individual* conditions. According to Löbner [1], *Individual* concepts possess a unique referent to every appropriate context of utterance. As *Individual* nouns are inherently unique, their lexical meanings are congruent with the concept type indicated by the definite article. If non-unique nouns are used with definite determination, their concepts are enriched with discourse context to meet the requirements of the *Individual* or *Functional* concept. According to Löbner [124], the cases where the referent of the definite article is established independently of the immediate situation or the context of an utterance are "semantic definites". The cases where the introduction of the referent of the definite article depends on the immediate situation and the discourse context are "pragmatic definites". The comparison of the congruent *Individual* and *Sortal* conditions in our study demonstrates the use of semantic and pragmatic definites, respectively. Whereas the requirement of a referent of a definite NP is saturated by the uniqueness in the case of semantically definite *Individual* items, there is no referent for the definite NP in the case of incongruent *Sortal* items, as there is no discourse context.

A study by Burkhardt [125], investigated the contrast between semantic definites (individual concepts, proper names, and indexicals) and pragmatic definites (definite NPs and third person pronouns dependent on discourse representation for reference specification) in a sentence context. The results of the study showed that context-dependent NPs triggered a more negative brain response at central electrodes, relative to inherently definite NPs. This effect was compatible with the latency and topography of N400 [126, 127]. Although the results of our studies revealed a difference between the IC and SI conditions at frontal and central midline sites, this effect had the opposite polarity: the discourse dependent definites, i.e. SI items, elicited a more positive brain response, relative to the semantic definites. The difference in the brain responses triggered by additional processing costs in our study and the study by Burkhardt [125] could be attributed to the disparities in experimental design and procedure. Burkhardt presented her stimuli visually embedded in sentences, whereas we employed an auditory presentation of noun phrases that were not embedded in any sentence or utterance.

A study by Schumacher [128] contrasted the processing of indefinite NPs with that of definite NPs in an utterance. A preamble introduced the context, based on which the NP in the following sentence could be perceived as given, inferred or new. The results showed that definite and indefinite NPs that were new in the discourse evoked an N400 effect. New and inferred definite NPs elicited a late positivity at parietal sites, whereas definite determiners in general triggered a LAN effect. Although we observed a congruence effect in the Sortal CT, i.e. a significant difference between congruent indefinite and incongruent definite determination, the polarity and the topographic distribution of this effect was distinct from the effects reported in the studies by Burkhardt [125] and Schumacher [128].

## Conclusion

The results of the present series of studies showed that the congruence effect depended on the concept type and on the inherent property of the concepts that was shifted, i.e. [U] or [R]. Since concept type incongruence elicited brain responses that were distinct from the classic semantic and syntactic components, the possibility that the processing of concept type shifts could be supported by the mechanisms underlying semantic or syntactic processing can be rejected. The concept-type specific incongruence responses that we observed need to be further investigated to assess their robustness and functional significance. Studies employing better source localization techniques, such as MEG or fMRI, are necessary in order to disentangle the neural underpinning of the concept type shift operation.
